# Author Correction: Risk-stratified treatment for drug-susceptible pulmonary tuberculosis

**DOI:** 10.1038/s41467-025-59791-2

**Published:** 2025-05-13

**Authors:** Vincent K. Chang, Marjorie Z. Imperial, Patrick P. J. Phillips, Gustavo E. Velásquez, Payam Nahid, Andrew Vernon, Ekaterina V. Kurbatova, Susan Swindells, Richard E. Chaisson, Susan E. Dorman, John L. Johnson, Marc Weiner, Amina Jindani, Thomas Harrison, Erin E. Sizemore, William Whitworth, Wendy Carr, Kia E. Bryant, Deron Burton, Kelly E. Dooley, Melissa Engle, Pheona Nsubuga, Andreas H. Diacon, Nguyen Viet Nhung, Rodney Dawson, Radojka M. Savic, AIDS Clinical Trial Group, William Whitworth, Wendy Carr, Deron Burton, Pheona Nsubuga, Rodney Dawson, Harriet Mayanja Kizza, Elias Ssaku, Isaac Sekitoleko, Joseph P. Akol, Andreas Diacon, Carmen Kleinhans, Julia Sims, Erika Mitchell, Bronwyn Hendricks, Yvetot Joseph, Marie Jude Jean Louis, Cadette Mercy, Alexandra Apollon, Gertrude Royal, Pamela Mukwekwerere, Yeukai Musodza, Wilfred Gurupira, Michele Tameris, Angelique Kany Kany Luabeya, Mark Hatherill, Mario Camblart, Circée Phara Jean, Mohammed Rassool, Noluthando Mwelase, Jaclyn Bennet, Lerato Mohapi, Ntebo Mogashoa, Debra Peters, Sanjay Gaikwad, Neetal Neverkar, Rahul Lokhande, Cornelius Munyanga, Mina Hosseinipour, Charity Potani, Elisha Okeyo, Samuel Gurrion Ouma, Prisca Rabuogi, Rodrigo Escada, Lidiane Tuler, Johnstone Kumwenda, Kelvin Mponda, Lynne Cornelissen, Andriette Hiemstra, Umesh G. Lalloo, Sandy Pillay, Abraham Siika, Alberto Mendoza, Pedro Gonzales, Mey Leon, Javier R. Lama, Alvaro Schwalb, Eduardo Gotuzzo, Fredrick Sawe, Isaac Tsikhutsu, Sivaporn Gatechompol, Anchalee Avihingsanon, Natthapol Kosashunhanan, Patcharaphan Sugandhavesa, Marineide Gonçalves de Melo, Rita de Cassia Alves Lira, Anne Luetkemeyer, Carina Marquez, Kristine Coughlin, Kelly E. Dooley, Jacques H. Grosset, Eric L. Nuermberger, Lara Hosey, Anthony T. Podany, Andrey Borisov, Nicole Brown, Scott Burns, Crystal Carter, Lauren Cowan, Melinda Dunn, Barbara DeCausey, Melissa Fagley, Kimberly Hedges, Constance Henderson, Amanda Hott, Carla Jeffries, Katherine Klein, Joan Mangan, Gerald Mazurek, Ruth Moro, Lakshmi Peddareddy, James Posey, Mary Reichler, Jessica Ricaldi, Claire Sadowski, Melisa Willby, Yan Yuan, April C. Pettit, Tuberculosis Trials Consortium, Lien T. Luu, Hanh T. T. Nguyen, Hung V. Nguyen, Hue T. M. Nguyen, Cyndy Merrifield, Matebogo Xaba, Maya Jaffer, Keitumetse Majoro, Kwok-Chiu Chang, Chi Chiu Leung, Polo Pavon, Rogelio Duque, George Samuel, Joseph Burzynski, Mascha Elskamp, Jill Campbell, Marlon Quintero, Elizabeth Guy

**Affiliations:** 1 Department of Bioengineering and Therapeutic Sciences, University of California San Francisco, San Francisco, CA USA; 2 UCSF Center for Tuberculosis, University of California San Francisco, San Francisco, CA USA; 3 Division of Pulmonary and Critical Care Medicine, University of California San Francisco, San Francisco, CA USA; 4 Division of HIV, Infectious Diseases, and Global Medicine, University of California San Francisco, San Francisco, CA USA; 5 Centers for Disease Control and Prevention, Atlanta, GA USA; 6 University of Nebraska Medical Center, Omaha, NE USA; 7 Center for Tuberculosis Research, Johns Hopkins University School of Medicine, Baltimore, MA USA; 8 Medical University of South Carolina, Charleston, SC USA; 9 Case Western Reserve University, University Hospitals Cleveland Medical Center, Cleveland, OH USA; 10 Uganda–Case Western Reserve University Research Collaboration, Kampala, Uganda; 11 University of Texas Health Science Center at San Antonio and the South Texas Veterans Health Care System, San Antonio, TX USA; 12 St. George’s, University of London, London, UK; 13 Division of Infectious Diseases, Vanderbilt University, Nashville, TN USA; 14 TASK, Cape Town, Western Cape, Cape Town, South Africa; 15 Vietnam National Tuberculosis Program–University of California, San Francisco Research Collaboration Unit, Hanoi, Vietnam; 16 National Lung Hospital, Hanoi, Vietnam; 17 Lung Institute and Division of Pulmonology, Department of Medicine, University of Cape Town, Cape Town, South Africa; 18 Les Centre GHESKIO INLR, Port au Prince, Port au Prince, Haiti; 19 Parirenyatwa Clinical Research Site, Harare, Zimbabwe; 20 South African Tuberculosis Vaccine Initiative (SATVI), Worcester, South Africa; 21 Les Centre GHESKIO IMIS, Port au Prince, Haiti; 22 Wits Helen Joseph Clinical Research Site Department of Medicine, Johannesburg, South Africa; 23 Soweto ACTG Clinical Research Site, Soweto, South Africa; 24 Byramjee Jeejeebhoy Medical College, Pune, India; 25 University of North Carolina Project Tidziwe Centre, Lilongwe, Malawi; 26 Kisumu Clinical Research Site, Kisumu, Kenya; 27 Institute de Pesquica Evandro Chagas Fiocruz, Rio de Janeiro, Brazil; 28 Blantyre Clinical Research Site, Johns Hopkins Research Project, Blantyre, Malawi; 29 Family Clinical Research Unit, University of Stellenbosch, Parow Valley, South Africa; 30 Durban International Clinical Research Site, Durban, South Africa; 31 Moi University Clinical Research Site, Eldoret, Kenya; 32 San Miguel Clinical Research Site, IMPACTAPERU, Putamayo, Peru; 33 Asociacion Civil Impacta Salud y Educacion, Lima, Peru; 34 Universidad Peruana Cayetano Heredia, San Martín de Porres, Lima, Peru; 35 Kenya Medical Research Institute/Walter Reed Project Clinical Research Center, Kericho, Kenya; 36 The Thai Red Cross AIDS Research Centre, Bangkok, Thailand; 37 HIV Treatment Clinical Research Site, Chiang Mai University, Chiang Mai, Thailand; 38 Hospital Conceicao, Porto Alegre, Brazil; 39 Frontier Sciences, Amherst, New York, NY USA; 40 Social and Scientific Systems, AIDS Clinical Trials Group Operation Center, New York, NY USA; 41 Vanderbilt University School of Medicine, Nashville, TN USA; 42 Wits Health Consortium Perinatal HIV Research Unit (PHRU), Johannesburg, South Africa; 43 Tuberculosis and Chest Service of Hong Kong, Hong Kong, China; 44 University of North Texas Health Science Center, Fort Worth, TX USA; 45 Columbia University, New York, NY USA; 46 Austin Tuberculosis Clinic, Austin, TX USA; 47 Baylor College of Medicine & Affiliated Hospitals/VA, Houston, TX USA

**Keywords:** Tuberculosis, Biomarkers, Clinical pharmacology, Pharmacokinetics, Pharmacodynamics

Correction to: *Nature Communications* 10.1038/s41467-024-53273-7, published online 30 October 2024

In the version of the article initially published, Amina Jindani and Thomas Harrison (both St. George’s, University of London, London, UK) were missing from the author list and have now been added. Additionally, the text “We thank Daniel Grint and the RIFASHORT team for providing us access to the data for external validation” was missing from the Acknowledgements section and has now been added. These corrections have been made to the HTML and PDF versions of the article.

