# Supplementary material for: Risk-stratified treatment for drug-susceptible pulmonary tuberculosis
Source: Nat Commun. 2024 Oct 30;15:9400. doi: 10.1038/s41467-024-53273-7 (PMC11526018; doi:10.1038/s41467-024-53273-7)
Supplement: Supplementary file 1 — Supplementary Information [file 41467_2024_53273_MOESM1_ESM.pdf]

Supplementary Methods

PK samples were taken between the 2 week and 8 week visit windows, after enzyme induction reached steady state[13]. Sparse PK samples were obtained from all participants at 0.5, 5, and 24 hours. Intensive PK samples were obtained from 53 participants with additional timepoints at 3, 9, and 12 hours.

The proportional hazards assumption for each covariate was evaluated by testing the correlation between Schoenfield residuals and time of event.[1–3] A correlation of zero indicates that the model met the proportional hazards assumption (the null hypothesis). Covariates that reject the null hypothesis with a significant p-value therefore violate the proportional hazards assumption.

As an additional pharmacokinetic-pharmacodynamic analysis, we compared rifapentine and rifapentine-moxifloxacin regimens dichotomized by median rifapentine exposure with Cox proportional hazards models. We performed Univariate and multivariable Cox proportional hazards analysis on demographic, baseline clinical, and pharmacokinetic factors as described in the main text. We performed a sensitivity analysis including and excluding imputed pharmacokinetic values from Univariate and multivariable analyses. We performed subgroup analyses of risk factors identified in multivariable analysis comparing risk differences dichotomized by the median value of each risk factor. TB-ReFLECT risk phenotype definitions[4] were assessed with Study 31/A5349 data by calculating risk differences and calculating the 95% Wald confidence interval.

We performed Univariate and multivariable logistic regression of any grade 3 or higher adverse events for participants receiving the rifapentine regimen (control and rifapentine-moxifloxacin regimen results are reported in the main text). We performed a sensitivity analysis including and excluding imputed pharmacokinetic values on Univariate and multivariable safety analyses.

Supplementary Results

Results of assessing proportional hazards assumption of all covariates by regimen are reported in Supplementary Tables 1-3. We reviewed each Kaplan-Meier curve by covariate individually when there was evidence of non-proportionality ($p < 0{\cdot}05$) but given the small number of events and since survival curves did not cross or diverge substantially, we felt comfortable continuing with these models.

Among participants with above-median rifapentine exposure, only two participants experienced tuberculosis (TB)-related unfavorable outcomes during the 4-month treatment period. Both participants were not seen at the 12-month follow-up visit and their last culture was positive during the treatment period. TB-related unfavorable rates were comparable across arms at 12 months post-randomization (rifapentine-moxifloxacin regimen: HR 0·86 relative to rifapentine regimen, 95% CI 0·42–1·75) (Supplementary Figure 8). In contrast, in participants with below-median rifapentine exposure, the substitution of moxifloxacin for ethambutol improved 12-month unfavorable outcomes from 14·5% in those who received the rifapentine regimen to 9·8% in those who received the rifapentine-moxifloxacin regimen (rifapentine-moxifloxacin regimen: HR 0·49 relative to rifapentine regimen, 95% CI 0·32–0·77). The main text of the manuscript demonstrated this finding stratified by regimen, risk group, and rifamycin exposure; it is reiterated here stratified by regimen and rifamycin exposure for emphasis.

Among participants receiving the rifapentine-moxifloxacin regimen, Univariate Cox proportional hazards analysis identified Black race (relative to Asian), lower Xpert MTB/RIF cycle threshold, lower rifapentine $AUC_{0–24h}$, lower rifapentine C_{max}, lower pyrazinamide $AUC_{0–24h}$, and lower isoniazid $AUC_{0–24h}$ as associated with increased hazard of TB-related unfavorable outcomes (threshold $P<0·05$, Supplementary Table 4). Among participants receiving the rifapentine regimen, factors associated with increased hazard of TB-related unfavorable outcomes on Univariate analysis included: older age, male sex, lower weight, lower BMI, lower Xpert MTB/RIF, shorter time to detection on sputum liquid culture, aggregate cavity size >4cm, extent of disease involvement of >50% thoracic cavity area on chest radiography, living with HIV, living with diabetes, history of liver disease, lower rifapentine $AUC_{0–24h}$, lower rifapentine C_{max}, lower ethambutol $AUC_{0–24h}$, lower ethambutol C_{max}, lower isoniazid $AUC_{0–24h}$, and lower isoniazid C_{max} (threshold $P<0·05$, Supplementary Table 5). Among participants receiving the control regimen, factors associated with increased hazard of TB-related unfavorable outcomes on Univariate analysis included: older age, lower Xpert MTB/RIF cycle threshold, current smoker (relative to nonsmoker), lower pyrazinamide $AUC_{0–24h}$, lower pyrazinamide C_{max}, and lower isoniazid C_{max} (threshold $P<0·05$, Supplementary Table 6). Multivariable results were presented in the main text.

Univariate and multivariable analyses were repeated excluding all imputed pharmacokinetic values. Findings were consistent with those reported in the main text for participants receiving the rifapentine-moxifloxacin and rifapentine regimens. For participants receiving the control regimen, pyrazinamide C_{max} was no longer associated with hazard of TB-related unfavorable outcomes after excluding imputed pharmacokinetic values, and in the multivariable model pyrazinamide $AUC_{0–24h}$ was also no longer associated with hazard (Supplementary Table 7 and 8).

Univariate Subgroup Analyses

The rifapentine-moxifloxacin regimen was noninferior to the control at the trial level. We therefore sought to identify high-risk subpopulations of participants that had large risk differences relative to control and for whom the rifapentine-moxifloxacin regimen might not be appropriate. Among participants who received the rifapentine-moxifloxacin regimen, those with ≥50% disease extent on chest radiography and those with low rifapentine exposure experienced higher TB-related unfavorable outcomes compared to the control (≥50% disease extent: risk difference 5·2%, 95% CI 1·9%–8·6%; low rifapentine exposure: risk difference 5·4%, 95% CI 2·4%–8·5%). Participants with an Xpert MTB/RIF cycle threshold of <18 had 3·5% risk difference of TB-related unfavorable outcomes when compared to the control, but the upper border of the 95% CI exceeded the 6·6% margin (95% CI, 0·4 – 6·7). All other subpopulations stratified by single risk factors (age and weight) had small risk differences. Rifapentine exposure had a significant interaction with regimen ($P < 0·03$), while no other interactions were significant (Supplementary Figure 4A).

The rifapentine regimen did not achieve noninferiority compared to the control at the trial level. We therefore sought to identify subpopulations of participants that had small risk differences relative to control and help define the low-risk subpopulations. Among participants receiving the rifapentine regimen, those with high rifapentine exposure had similar rates of TB-related unfavorable outcomes compared to the control group (risk difference 0·5%, 95% CI −2·2%–3·2%). Participants with an Xpert MTB/RIF cycle threshold of ≥18 and those with <50% disease extent on chest radiography also had similar rates of TB-related unfavorable outcomes at 12 months across the rifapentine and control regimens (Xpert MTB/RIF cycle threshold ≥18: risk difference 2·7%, 95% CI −0·2%–5·6%; <50% disease extent: risk difference 4·1%, 95% CI 1·4%–6·8%). All other subpopulations stratified by single risk factors (age and weight) had larger risk differences or wide confidence intervals. Rifapentine exposure had a significant interaction with regimen ($P < 0·001$), while no other interactions were significant (Supplementary Figure 4B).

Prespecified Risk Phenotype Validation

We assessed prespecified disease phenotype definitions in the TB-ReFLECT analysis by Imperial et al.;[4] whereby easier-to-treat TB defined as sputum AFB smear grade <2 or noncavitary disease had similar rates of TB-related unfavorable outcome across the experimental and control regimens, and in harder-to-treat TB, defined as sputum AFB smear grade ≥3 and cavitary disease, the experimental group experienced higher TB-related unfavorable outcomes than the control. For those receiving rifapentine-moxifloxacin regimen, TB-ReFLECT defined easier-to-treat TB had similar rates of TB-related unfavorable outcome across the experimental and control regimens (easier-to-treat TB: risk difference 2·8%, 95%

CI 0%–5·5%). Harder-to-treat TB defined by the TB-ReFLECT analysis also experienced similar rates of tuberculosis-related unfavorable outcome, however the upper bound of the confidence interval was beyond the 6·6% margin (risk difference 2·2%, 95% CI −2·2%–6·7%) (Supplementary Figure 6A).

For those receiving rifapentine regimen, participants classified as having easier-to-treat TB by the TB-ReFLECT definition had a 4% risk difference compared to the control, however the upper bound of the confidence interval was just beyond the 6·6% margin (risk difference 4·0%, 95% CI 1·1%–6·9%); participants classified as having harder-to-treat TB by the TB-ReFLECT definition had a large risk difference compared to the control (risk difference 10·9%, 95% CI 5%–16·7%) (Supplementary Figure 6B).

There were only 5·7% TB-related unfavorable outcomes in the rifapentine-moxifloxacin regimen. Sputum AFB smear grade and presence of cavitation are lower resolution measurements than Xpert MTB/RIF cycle threshold[5] and disease extent on chest radiograph. The more potent noninferior rifapentine-moxifloxacin regimen may need finer measurements to tease out harder-to-treat TB. Finally, the TB-ReFLECT risk strata were defined from regimens that all failed to achieve noninferiority (OFLOTUB[6], ReMOX[7], RIFAQUIN[8]), we therefore see a clear validation of the TB-ReFLECT risk phenotypes in the rifapentine regimen, which is a more similar comparison to TB-ReFLECT regimens, while no gradient response in TB-ReFLECT risk phenotypes receiving the rifapentine-moxifloxacin regimen. The advantages to the TB-ReFLECT phenotypes are implementation in settings without access to Xpert MTB/RIF, although access to Xpert is becoming more widespread.

Safety

Among participants receiving the rifapentine regimen, Univariate logistic regression found older age, Asian race (relative to Black), non-African clinical site (relative to African), history of liver disease, and higher ethambutol exposure to be associated with risk of any grade 3 or higher adverse events (threshold $P<0·05$, Supplementary Table 12). Multivariable analysis the following factors to be associated with risk of any grade 3 or higher adverse events: Asian race (OR 2·09 relative to Black race, 95% CI 1·19–3·56) and ethambutol $AUC_{0–24h}$ (OR 1·38 for every 5 µg·h/mL increase, 95% CI 1·01–1·95).

We repeated Univariate and multivariable analyses excluding all imputed pharmacokinetic values, with findings mostly consistent with those reported in the main text. For participants receiving the rifapentine-moxifloxacin regimen, Univariate

sensitivity analysis excluding imputed pharmacokinetic values were consistent with the main analysis. In multivariable sensitivity analysis excluding imputed pharmacokinetic values, history of liver disease was no longer significantly associated with any grade 3 or higher adverse events. For participants receiving the rifapentine regimen, in the main analysis ethambutol AUC_{0-24h} was not associated with any grade 3 or higher adverse events but was found to be significantly associated in the sensitivity analysis excluding imputed pharmacokinetic values. In multivariable sensitivity analysis excluding imputed pharmacokinetic values, history of liver disease was no longer associated with any grade 3 or higher adverse events. For participants receiving the control regimen, in the main analysis ethambutol C_{max} was not associated with any grade 3 or higher adverse events but was associated in the sensitivity analysis excluding imputed pharmacokinetic values. In multivariable sensitivity analysis excluding imputed pharmacokinetic values findings were consistent with those reported in the main analysis and text (Supplementary Table 13 and 14).

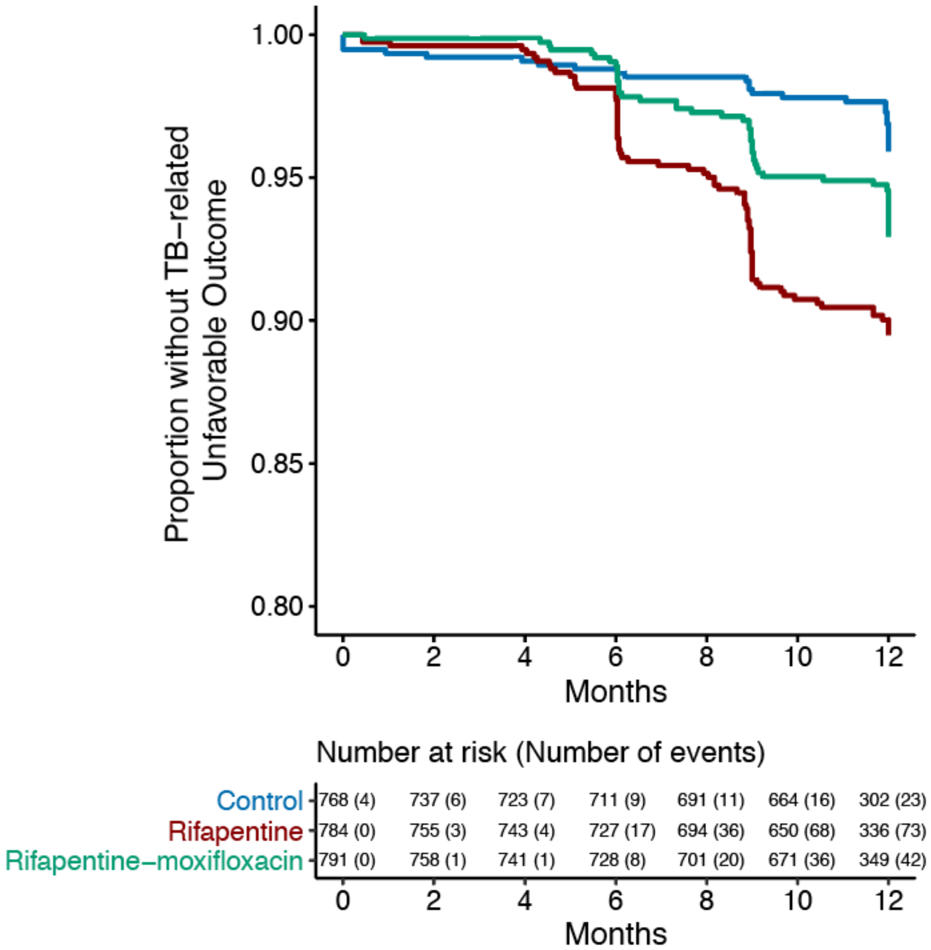

Supplementary Figure 1. Kaplan-Meier Estimates of Time to Tuberculosis-Related Unfavorable Outcomes.

Favorable outcomes and not tuberculosis-related unfavorable outcomes were right-censored at the time of last visit and time to event.

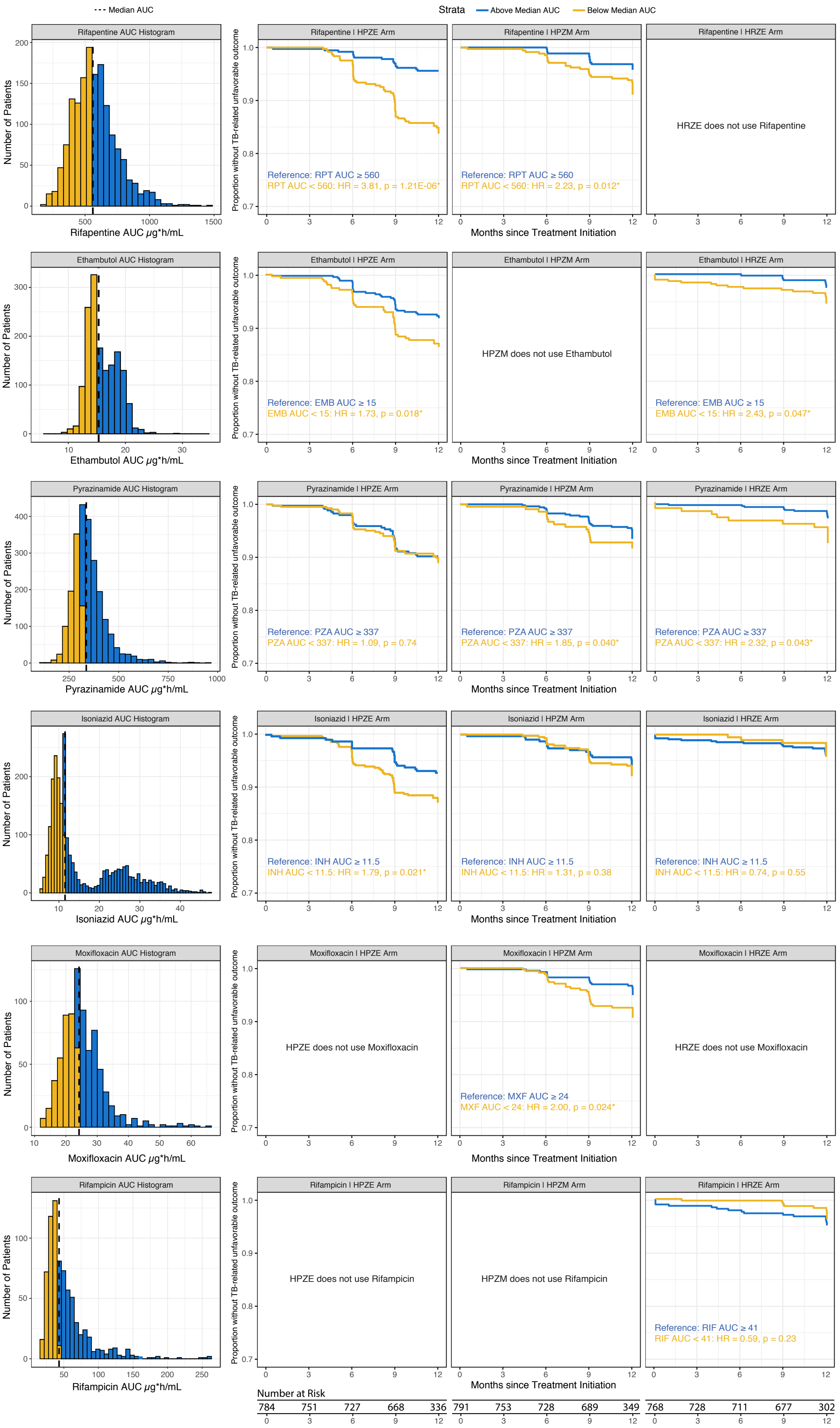

Median AUC
Strata
Above Median AUC
Below Median AUC
Rifapentine AUC Histogram
Number of Patients
Rifapentine AUC μg*h/mL
Rifapentine | HPZE Arm
Rifapentine | HPZM Arm
Rifapentine | HRZE Arm
Proportion without TB-related unfavorable outcome
Reference: RPT AUC ≥ 560
RPT AUC < 560: HR = 3.81, p = 1.21E-06*
Reference: RPT AUC ≥ 560
RPT AUC < 560: HR = 2.23, p = 0.012*
HRZE does not use Rifapentine
Months since Treatment Initiation
Ethambutol AUC Histogram
Ethambutol AUC μg*h/mL
Ethambutol | HPZE Arm
Ethambutol | HPZM Arm
Ethambutol | HRZE Arm
Reference: EMB AUC ≥ 15
EMB AUC < 15: HR = 1.73, p = 0.018*
HPZM does not use Ethambutol
Reference: EMB AUC ≥ 15
EMB AUC < 15: HR = 2.43, p = 0.047*
Pyrazinamide AUC Histogram
Pyrazinamide AUC μg*h/mL
Pyrazinamide | HPZE Arm
Pyrazinamide | HPZM Arm
Pyrazinamide | HRZE Arm
Reference: PZA AUC ≥ 337
PZA AUC < 337: HR = 1.09, p = 0.74
Reference: PZA AUC ≥ 337
PZA AUC < 337: HR = 1.85, p = 0.040*
Reference: PZA AUC ≥ 337
PZA AUC < 337: HR = 2.32, p = 0.043*
Isoniazid AUC Histogram
Isoniazid AUC μg*h/mL
Isoniazid | HPZE Arm
Isoniazid | HPZM Arm
Isoniazid | HRZE Arm
Reference: INH AUC ≥ 11.5
INH AUC < 11.5: HR = 1.79, p = 0.021*
Reference: INH AUC ≥ 11.5
INH AUC < 11.5: HR = 1.31, p = 0.38
Reference: INH AUC ≥ 11.5
INH AUC < 11.5: HR = 0.74, p = 0.55
Moxifloxacin AUC Histogram
Moxifloxacin AUC μg*h/mL
Moxifloxacin | HPZE Arm
Moxifloxacin | HPZM Arm
Moxifloxacin | HRZE Arm
HPZE does not use Moxifloxacin
Reference: MXF AUC ≥ 24
MXF AUC < 24: HR = 2.00, p = 0.024*
HRZE does not use Moxifloxacin
Rifampicin AUC Histogram
Rifampicin AUC μg*h/mL
Rifampicin | HPZE Arm
Rifampicin | HPZM Arm
Rifampicin | HRZE Arm
HPZE does not use Rifampicin
HPZM does not use Rifampicin
Reference: RIF AUC ≥ 41
RIF AUC < 41: HR = 0.59, p = 0.23
Number at Risk
784 751 727 668 336
791 753 728 689 349
768 728 711 677 302

Supplementary Figure 2. Steady State $AUC_{0–24h}$ Histograms and Kaplan Meier Estimates of Time to Tuberculosis-Related Unfavorable Outcomes Stratified by Arm and Drug Exposure. Hazard ratios and p-values for the log-rank test are reported in each plot.

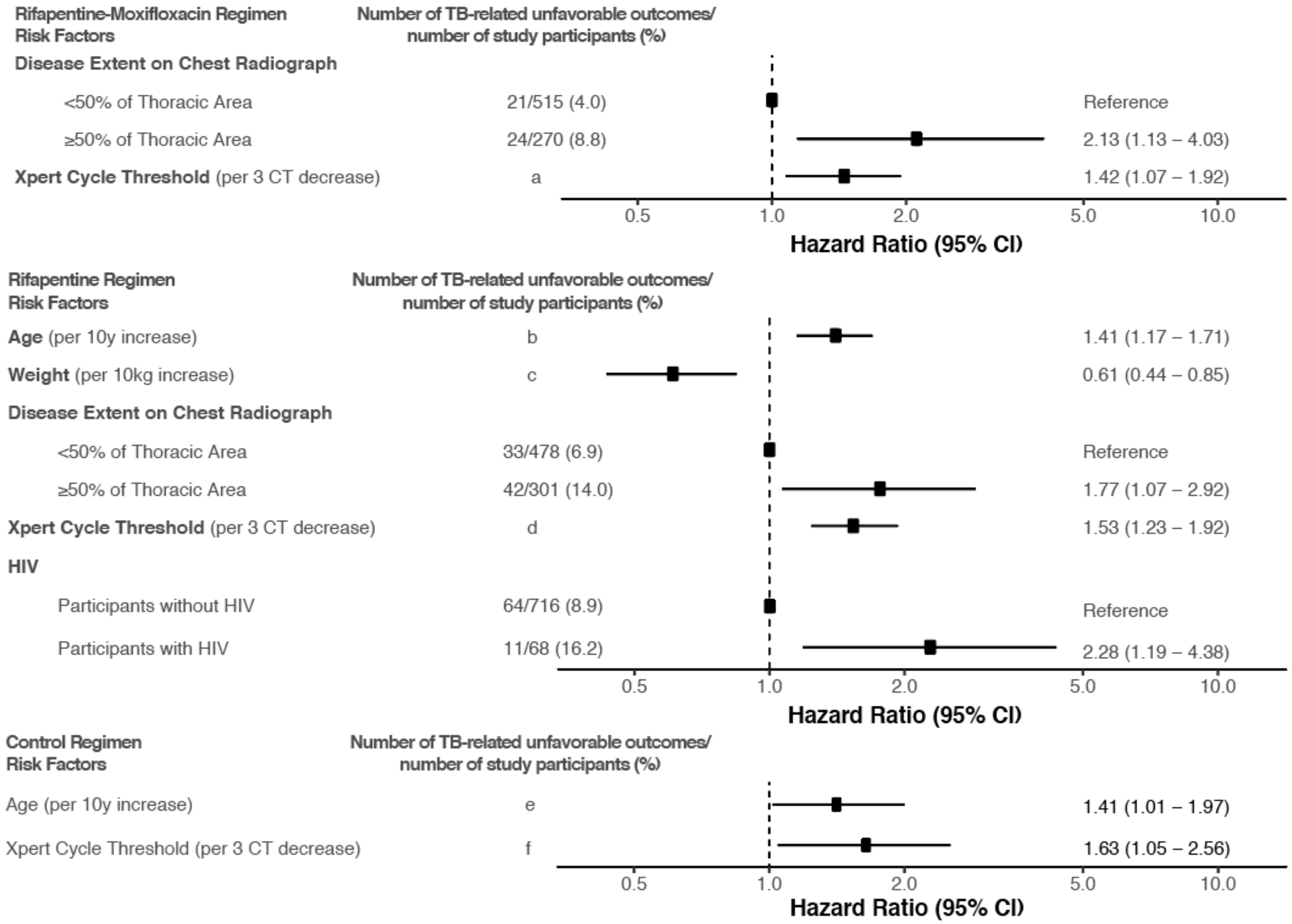

Supplementary Figure 3. Multivariable Hazard Ratios for Tuberculosis-Related Unfavorable Outcomes with Only Baseline Predictors. Multivariable analysis of baseline predictors (without pharmacokinetic predictors) for the rifapentine-moxifloxacin, rifapentine, and control regimens. **(a)** Xpert MTB/RIF cycle threshold < 18, 29/397 (7·3); Xpert MTB/RIF cycle threshold ≥ 18, 10/296 (3·4), **(b)** Age < 30 years, 21/354 (5·9); Age ≥ 30 years, 54/430 (12·6), **(c)** Weight < 53 kg, 45/364 (12·4); Weight ≥ 53 kg, 30/419 (7·2), **(d)** Xpert MTB/RIF cycle threshold < 18, 54/397 (13·6); Xpert MTB/RIF cycle threshold ≥ 18, 13/284 (7·7), **(e)** Age < 30 years, 4/353 (1·1); Age ≥ 30 years, 20/415 (4·8), **(f)** Xpert MTB/RIF cycle threshold < 18, 15/399 (3·7); Xpert MTB/RIF cycle threshold ≥ 18, 5/268 (1·9).

Supplementary Table 1. Assessment of Cox proportional hazards assumption for all potential covariates in the Rifapentine-Moxifloxacin Arm. Tests of correlation between the Schoenfield residuals and survival time were performed for each covariate. A correlation of zero indicates that the model met the proportional hazards assumption (the null hypothesis). Covariates (bolded) that reject the null hypothesis with a significant two-tailed Chi-squared p-value therefore violate the proportional hazards assumption.

Predictor	Rho	Chi Squared	p-value
DEMOGRAPHIC FACTORS			
Age	0·20	1·59	0·21
Sex	-0·14	0·84	0·36
WT	-0·19	1·66	0·20
**BMI**	**-0·37**	**5·05**	**0·025**
Black Race (relative to Mixed)	-0·10	0·44	0·51
Asian Race (relative to Mixed)	-0·05	0·12	0·73
African clinical site (relative to non-African)	-0·19	1·56	0·21
BASELINE CLINICAL FACTORS			
**Xpert MTB/RIF CT**	**-0·38**	**5·81**	**0·016**
**Time to Detection on Sputum Liquid Culture**	**-0·39**	**14·20**	**0·00164**
Presence of Cavitation	0·20	1·73	0·19
Cavity Class >=4 cm (relative to <4cm/no cavities)	0·22	2·14	0·14
Extent of disease (>50% relative to <25%/25–50%)	0·15	1·05	0·31
Smear grade 0 relative to 2	0·04	0·00	1·00
Smear grade 0·5 relative to 2	0·24	2·54	0·11
Smear grade 1 relative to 2	-0·12	0·70	0·40
Smear grade 3 relative to 2	0·05	0·10	0·75
Karnofsky score	-0·01	0·00	0·95
Living with HIV (relative to without HIV)	0·18	1·54	0·21
History of Diabetes (relative to no history)	0·06	0·15	0·70
Smoking history (former relative to nonsmoker)	0·02	0·01	0·90
Smoking history (current relative to nonsmoker)	-0·15	0·99	0·32
History of Liver Disease	-0·20	0·00	1·00
PHARMACOKINETIC FACTORS			
Rifapentine $AUC_{0–24h}$	0·10	0·45	0·50
Rifapentine C_{max}	0·22	1·83	0·18
Ethambutol $AUC_{0–24h}$	-0·07	0·41	0·52
Ethambutol C_{max}	-0·11	0·72	0·40
Pyrazinamide $AUC_{0–24h}$	-0·02	0·02	0·90
Pyrazinamide C_{max}	-0·07	0·27	0·60
Isoniazid $AUC_{0–24h}$	0·15	1·12	0·29
Isoniazid C_{max}	0·20	1·59	0·21

Supplementary Table 2. Assessment of Cox proportional hazards assumption for all potential covariates in the Rifapentine Arm. Tests of correlation between the Schoenfield residuals and survival time were performed for each covariate. A correlation of zero indicates that the model met the proportional hazards assumption (the null hypothesis). Covariates (bolded) that reject the null hypothesis with a significant two-tailed Chi-squared p-value therefore violate the proportional hazards assumption.

Predictor	Rho	Chi Squared	p-value
DEMOGRAPHIC FACTORS			
Age	-0·11	0·73	0·39
Sex	-0·18	2·33	0·13
WT	-0·04	0·10	0·76
BMI	-0·15	2·69	0·10
Black Race (relative to Mixed)	-0·03	0·08	0·78
**Asian Race (relative to Mixed)**	**-0·37**	**10·06**	**0·0015**
**African clinical site (relative to non-African)**	**-0·51**	**19·50**	**0·0000101**
BASELINE CLINICAL FACTORS			
Xpert MTB/RIF CT	0·01	0·01	0·91
Time to Detection on Sputum Liquid Culture	-0·08	0·57	0·45
**Presence of Cavitation**	**0·26**	**4·97**	**0·026**
**Cavity Class >=4 cm (relative to <4cm/no cavities)**	**0·23**	**4·08**	**0·043**
**Extent of disease (>50% relative to <25%/25–50%)**	**0·30**	**6·85**	**0·0089**
Smear grade 0 relative to 2	0·07	0·42	0·52
Smear grade 0·5 relative to 2	0·10	0·71	0·40
Smear grade 1 relative to 2	-0·04	0·11	0·74
Smear grade 3 relative to 2	0·14	1·35	0·25
Karnofsky score	-0·09	0·60	0·44
Living with HIV (relative to without HIV)	0·13	1·31	0·25
History of Diabetes (relative to no history)	-0·12	1·02	0·31
Smoking history (former relative to nonsmoker)	-0·03	0·06	0·81
Smoking history (current relative to nonsmoker)	-0·20	3·12	0·08
History of Liver Disease	-0·05	0·17	0·68
PHARMACOKINETIC FACTORS			
Rifapentine $AUC_{0–24h}$	-0·23	3·07	0·08
Rifapentine C_{max}	-0·23	3·28	0·07
Ethambutol $AUC_{0–24h}$	0·04	0·11	0·74
Ethambutol C_{max}	-0·03	0·07	0·78
Pyrazinamide $AUC_{0–24h}$	0·14	1·31	0·25
Pyrazinamide C_{max}	-0·09	0·46	0·50
Isoniazid $AUC_{0–24h}$	0·06	0·27	0·61
Isoniazid C_{max}	-0·11	0·73	0·39

Supplementary Table 3. Assessment of Cox proportional hazards assumption for all potential covariates in the Control Arm. Tests of correlation between the Schoenfield residuals and survival time were performed for each covariate. A correlation of zero indicates that the model met the proportional hazards assumption (the null hypothesis). Covariates (bolded) that reject the null hypothesis with a significant two-tailed Chi-squared p-value therefore violate the proportional hazards assumption.

**Predictor**	**Rho**	**Chi Squared**	**p-value**
DEMOGRAPHIC FACTORS			
Age	0·31	1·31	0·25
Sex	-0·35	2·90	0·09
WT	0·28	1·01	0·31
BMI	0·11	0·21	0·64
**Black Race (relative to Mixed)**	**0·72**	**12·40**	**0·000429**
**Asian Race (relative to Mixed)**	**0·45**	**4·80**	**0·028**
African clinical site (relative to non-African)	-0·35	2·83	0·09
BASELINE CLINICAL FACTORS			
Xpert MTB/RIF CT	-0·25	1·16	0·28
Time to Detection on Sputum Liquid Culture	-0·16	0·95	0·33
Presence of Cavitation	0·39	3·50	0·06
Cavity Class >=4 cm (relative to <4cm/no cavities)	0·04	0·04	0·83
Extent of disease (>50% relative to <25%/25–50%)	0·15	0·57	0·45
Smear grade 0 relative to 2	0·26	0·00	1·00
Smear grade 0·5 relative to 2	0·01	0·00	0·95
Smear grade 1 relative to 2	0·22	1·20	0·27
Smear grade 3 relative to 2	0·08	0·16	0·69
Karnofsky score	0·39	3·32	0·07
Living with HIV (relative to without HIV)	0·13	0·39	0·53
History of Diabetes (relative to no history)	0·16	0·64	0·42
Smoking history (former relative to nonsmoker)	0·12	0·36	0·55
Smoking history (current relative to nonsmoker)	-0·06	0·08	0·78
History of Liver Disease	-0·17	0·00	1·00
PHARMACOKINETIC FACTORS			
Rifapentine $AUC_{0–24h}$	-0·35	1·54	0·21
Rifapentine C_{max}	-0·29	0·96	0·33
Ethambutol $AUC_{0–24h}$	0·27	1·58	0·21
Ethambutol C_{max}	0·30	1·95	0·16
Pyrazinamide $AUC_{0–24h}$	0·21	1·09	0·30
Pyrazinamide C_{max}	0·08	0·20	0·66
Isoniazid $AUC_{0–24h}$	0·12	0·42	0·52
Isoniazid C_{max}	0·31	1·31	0·25

Supplementary Table 4. Unadjusted and Adjusted Hazard Ratios for Tuberculosis-Related Unfavorable Outcomes Among Participants Receiving the Rifapentine-Moxifloxacin Regimen. Hazard ratios, confidence intervals and two-tailed p-values calculated by Cox proportional hazards regression.

Predictor	Unadjusted Hazard Ratio	Unadjusted 95% CI	Unadjusted p-value	Adjusted Hazard Ratio	Adjusted 95% CI	Adjusted p-value
DEMOGRAPHIC FACTORS						
Age (for every 10-year increase)	1·08	0·85 – 1·37	0·55	--	--	--
Male sex (relative to female)	0·69	0·34 – 1·40	0·31	--	--	--
WT (for every 10-kg increase)	0·89	0·64 – 1·25	0·51	--	--	--
BMI (for every 1-unit increase)	0·95	0·86 – 1·06	0·36	--	--	--
**Black Race (relative to Mixed)**	**0·47**	**0·24 – 0·94**	**0·034**	--	--	--
Asian Race (relative to Mixed)	0·54	0·19 – 1·55	0·25	--	--	--
African clinical site (relative to non-African)	0·89	0·45 – 1·75	0·73	--	--	--
BASELINE CLINICAL FACTORS						
**Xpert MTB/RIF CT (for every 3 CT decrease)**	**1·47**	**1·10 – 1·97**	**0·00988**	**1·43**	**1·07 - 1·91**	**0·015**
Time to Detection on Sputum Liquid Culture (for every 1-day increase)	0·99	0·92 – 1·07	0·88	--	--	--
Presence of Cavitation	0·93	0·49 – 1·78	0·83	--	--	--
Cavity Class ≥4 cm (relative to <4cm/no cavities)	1·61	0·90 – 2·88	0·11	--	--	--
**Extent of disease (≥50% relative to <25%/25-50%)**	**2·23**	**1·24 – 4·01**	**0·0073**	**2·03**	**1·08 - 3·83**	**0·029**
Smear grade 0 relative to 2	0	0 – Inf	0·99	--	--	--
Smear grade 0·5 relative to 2	1·04	0·45 – 2·40	0·93	--	--	--
Smear grade 1 relative to 2	1·04	0·46 – 2·35	0·92	--	--	--
Smear grade 3 relative to 2	0·98	0·45 – 2·12	0·96	--	--	--
Karnofsky score (for every 10)	0·74	0·49 – 1·12	0·16	--	--	--
Living with HIV (relative to without HIV)	0·83	0·26 – 2·67	0·75	--	--	--
History of Diabetes (relative to no history)	0·55	0·08 – 3·98	0·55	--	--	--
Smoking history (former relative to nonsmoker)	0·59	0·29 – 1·20	0·15	--	--	--
Smoking history (current relative to nonsmoker)	1·08	0·50 – 2·34	0·84	--	--	--
History of liver disease	0	0 – Inf	0·99	--	--	--
PHARMACOKINETIC FACTORS						
**Rifapentine $AUC_{0–24h}$ (for every 100 μg·h/mL)**	**0·77**	**0·64 – 0·93**	**0·00648**	**0·77**	**0·63 - 0·95**	**0·015**
**Rifapentine C_{max} (for every 10 μg/mL)**	**0·77**	**0·63 – 0·93**	**0·00828**	--	--	--
Moxifloxacin $AUC_{0–24h}$ (for every 5 μg·h/mL)	0·82	0·64 – 1·05	0·12	--	--	--
Moxifloxacin C_{max} (for every 1 μg/mL)	0·78	0·49 – 1·24	0·29	--	--	--
**Pyrazinamide $AUC_{0–24h}$ (for every 100 μg·h/mL)**	**0·60**	**0·40 – 0·91**	**0·016**	--	--	--
Pyrazinamide C_{max} (for every 10 μg/mL)	0·63	0·38 – 1·06	0·080	--	--	--
**Isoniazid $AUC_{0–24h}$ (for every 5 μg·h/mL)**	**0·78**	**0·62 – 0·98**	**0·033**	--	--	--
Isoniazid C_{max} (for every 1 μg/mL)	0·82	0·54 – 1·24	0·34	--	--	--

Supplementary Table 5. Unadjusted and Adjusted Hazard Ratios for Tuberculosis-Related Unfavorable Outcomes Among Participants Receiving the Rifapentine Regimen. Hazard ratios, confidence intervals and two-tailed p-values calculated by Cox proportional hazards regression.

Predictor	Unadjusted Hazard Ratio	Unadjusted 95% CI	Unadjusted p-value	Adjusted Hazard Ratio	Adjusted 95% CI	Adjusted p-value
DEMOGRAPHIC FACTORS						
**Age (for every 10-year increase)**	**1·45**	**1·22 – 1·71**	**0·0000013**	**1·37**	**1·13 - 1·67**	**0·00166**
**Male sex (relative to female)**	**0·38**	**0·20 – 0·74**	**0·0044**			
**WT (for every 10-kg increase)**	**0·61**	**0·44 – 0·83**	**0·00152**	**0·57**	**0·40 - 0·80**	**0·00124**
**BMI (for every 1-unit increase)**	**0·86**	**0·79 – 0·95**	**0·00208**			
Black Race (relative to Mixed)	1·49	0·68 – 3·26	0·32			
Asian Race (relative to Mixed)	1·54	0·57 – 4·14	0·39			
African clinical site (relative to non-African)	0·58	0·32 – 1·05	0·072			
BASELINE CLINICAL FACTORS						
**Xpert MTB/RIF CT (for every 3 CT decrease)**	**1·63**	**1·32 – 2·02**	**0.0000062**	**1·54**	**1·93 - 1·24**	**0·00012**
**Time to Detection on Sputum Liquid Culture (for every 1-day increase)**	**0·90**	**0·83 – 0·97**	**0·00515**			
Presence of Cavitation	1·23	0·72 – 2·08	0·45			
**Cavity Class ≥4 cm (relative to <4cm/no cavities)**	**1·67**	**1·06 – 2·64**	**0·026**			
**Extent of disease (≥50% relative to <25%/25-50%)**	**2·09**	**1·32 – 3·29**	**0·00156**	**1·61**	**0·98 - 2·65**	**0·060**
Smear grade 0 relative to 2	0·28	0·04 – 2·04	0·21			
Smear grade 0·5 relative to 2	0·37	0·14 – 0·96	0·042			
Smear grade 1 relative to 2	0·65	0·33 – 1·29	0·22			
Smear grade 3 relative to 2	1·42	0·84 – 2·40	0·20			
Karnofsky score (for every 10)	0·93	0·66 – 1·31	0·66			
**Living with HIV (relative to without HIV)**	**1·95**	**1·03 – 3·71**	**0·040**			
**History of Diabetes (relative to no history)**	**6·53**	**2·83 – 15·1**	**0.0000105**			
**Smoking history (former relative to nonsmoker)**	**0·52**	**0·30 – 0·91**	**0·023**			
Smoking history (current relative to nonsmoker)	1·00	0·56 – 1·77	0·99			
**History of Liver Disease**	**5·27**	**1·29 – 21·5**	**0·020**			
PHARMACOKINETIC FACTORS						
**Rifapentine $AUC_{0–24h}$ (for every 100 μg·h/mL)**	**0·65**	**0·55 – 0·76**	**0.0000002**	**0·65**	**0·54 - 0·77**	**0.00000053**
**Rifapentine C_{max} (for every 10 μg/mL)**	**0·62**	**0·52 – 0·74**	**0.00000005**			
**Ethambutol $AUC_{0–24h}$ (for every 5 μg·h/mL)**	**0·54**	**0·33 – 0·86**	**0·00983**			
**Ethambutol C_{max} (for every 1 μg/mL)**	**0·56**	**0·34 – 0·92**	**0·022**			
Pyrazinamide $AUC_{0–24h}$ (for every 100 μg·h/mL)	0·87	0·64 – 1·19	0·38			
Pyrazinamide C_{max} (for every 10 μg/mL)	0·60	0·35 – 1·02	0·060			
**Isoniazid $AUC_{0–24h}$ (for every 5 μg·h/mL)**	**0·80**	**0·67 – 0·95**	**0·011**			
**Isoniazid C_{max} (for every 1 μg/mL)**	**0·53**	**0·35 – 0·81**	**0·00295**			

Supplementary Table 6. Unadjusted and Adjusted Hazard Ratios for Tuberculosis-Related Unfavorable Outcomes Among Participants Receiving the Control Regimen. Hazard ratios, confidence intervals and two-tailed p-values calculated by Cox proportional hazards regression.

Predictor	Unadjusted Hazard Ratio	Unadjusted 95% CI	Unadjusted p-value	Adjusted Hazard Ratio	Adjusted 95% CI	Adjusted p-value
**DEMOGRAPHIC FACTORS**						
**Age (for every 10-year increase)**	**1·41**	**1·05 – 1·90**	**0·023**			
Male sex (relative to female)	1·45	0·63 – 3·30	0·38			
WT (for every 10-kg increase)	0·64	0·37 - 1·11	0·11			
BMI (for every 1-unit increase)	0·88	0·75 – 1·04	0·13			
Black Race (relative to Mixed)	0·71	0·24 – 2·09	0·53			
Asian Race (relative to Mixed)	0·49	0·09 – 2·70	0·42			
African clinical site (relative to non–African)	0·97	0·39 – 2·45	0·95			
**BASELINE CLINICAL FACTORS**						
**Xpert MTB/RIF CT (for every 3 CT decrease)**	**1·66**	**1·07 – 2·57**	**0·024**	**1·69**	**1·08 2·63**	**0·021**
Time to Detection on Sputum Liquid Culture (for every 1-day increase)	0·98	0·88 – 1·09	0·72			
Presence of Cavitation	1·34	0·50 – 3·58	0·56			
Cavity Class ≥4 cm (relative to <4cm/no cavities)	1·46	0·66 – 3·26	0·35			
Extent of disease (≥50% relative to <25%/25-50%)	1·29	0·58 – 2·88	0·53			
Smear grade 0 relative to 2	0	0 – Inf	1			
Smear grade 0·5 relative to 2	0·78	0·21 – 2·94	0·71			
Smear grade 1 relative to 2	1·13	0·41 – 3·13	0·81			
Smear grade 3 relative to 2	0·88	0·31 – 2·54	0·82			
Karnofsky score (for every 10)	0·73	0·42 – 1·27	0·27			
Living with HIV (relative to without HIV)	0·53	0·07 – 3·94	0·54			
History of Diabetes (relative to no history)	2·38	0·56 – 10·1	0·24			
Smoking history (former relative to nonsmoker)	2·51	0·56 – 11·3	0·23			
**Smoking history (current relative to nonsmoker)**	**5·26**	**1·16 – 23·7**	**0·031**			
History of liver disease	0	0 – Inf	1			
**PHARMACOKINETIC FACTORS**						
Rifampicin $AUC_{0–24h}$ (for every 10 µg·h/mL)	1·02	0·93 - 1·11	0·67			
Rifampicin C_{max} (for every 1 µg/mL)	1·02	0·95 – 1·10	0·63			
Ethambutol $AUC_{0–24h}$ (for every 5 µg·h/mL)	1·09	0·91 – 1·30	0·37			
Ethambutol C_{max} (for every 1 µg/mL)	1·10	0·60 – 2·03	0·75			
**Pyrazinamide $AUC_{0—24h}$ (for every 100 µg·h/mL)**	**0·38**	**0·17 – 0·82**	**0·013**	**0·36**	**0·15 - 0·83**	**0·016**
**Pyrazinamide C_{max} (for every 10 µg/mL)**	**0·35**	**0·14 – 0·90**	**0·029**			
Isoniazid $AUC_{0–24h}$ (for every 5 µg·h/mL)	0·49	0·20 – 1·16	0·10			
**Isoniazid C_{max} (for every 1 µg/mL)**	**0·34**	**0·12 – 0·96**	**0·041**			

Supplementary Table 7. Sensitivity Analysis of Univariate Cox Proportional Hazards of Tuberculosis-Related Unfavorable Outcomes by Pharmacokinetic Factors, Including and Excluding Imputed Values. Hazard ratios, confidence intervals and two-tailed p-values calculated by Cox proportional hazards regression.

**Rifapentine-Moxifloxacin Regimen**	**Main Analysis Including Imputed PK**			**Sensitivity Analysis Excluding Imputed PK**		
**Predictor**	Unadjusted Hazard Ratio	Unadjusted 95% CI	Unadjusted p-value	Unadjusted Hazard Ratio	Unadjusted 95% CI	Unadjusted p-value
**PHARMACOKINETIC FACTORS**						
**Rifapentine $AUC_{0–24h}$ (for every 100 µg·h/mL)**	**0·77**	**0·64 – 0·93**	**0·00648**	**0·76**	**0·63 - 0·93**	**0·00697**
**Rifapentine C_{max} (for every 10 µg/mL)**	**0·77**	**0·63 – 0·93**	**0·00828**	**0·77**	**0·63 - 0·94**	**0·011**
Moxifloxacin $AUC_{0–24h}$ (for every 5 µg·h/mL)	0·82	0·64 – 1·05	0·12	0·82	0·63 - 1·06	0·13
Moxifloxacin C_{max} (for every 1 µg/mL)	0·78	0·49 – 1·24	0·29	0·80	0·49 - 1·30	0·37
**Pyrazinamide $AUC_{0–24h}$ (for every 100 µg·h/mL)**	**0·60**	**0·40 – 0·91**	**0·016**	**0·59**	**0·39 - 0·91**	**0·017**
Pyrazinamide C_{max} (for every 10 µg/mL)	0·63	0·38 – 1·06	0·080	0·61	0·36 - 1·04	0·069
**Isoniazid $AUC_{0–24h}$ (for every 5 µg·h/mL)**	**0·78**	**0·62 – 0·98**	**0·033**	**0·78**	**0·62 - 0·98**	**0·033**
Isoniazid C_{max} (for every 1 µg/mL)	0·82	0·54 – 1·24	0·34	0·82	0·54 - 1·24	0·34

**Rifapentine Regimen**	**Main Analysis Including Imputed PK**			**Sensitivity Analysis Excluding Imputed PK**		
**Predictor**	Unadjusted Hazard Ratio	Unadjusted 95% CI	Unadjusted p-value	Unadjusted Hazard Ratio	Unadjusted 95% CI	Unadjusted p-value
**PHARMACOKINETIC FACTORS**						
**Rifapentine $AUC_{0–24h}$ (for every 100 µg·h/mL)**	**0·65**	**0·55 – 0·76**	**0.0000002**	**0·64**	**0·54 - 0·76**	**0.00000015**
**Rifapentine C_{max} (for every 10 µg/mL)**	**0·62**	**0·52 – 0·74**	**0.00000005**	**0·61**	**0·51 - 0·73**	**0.00000004**
**Ethambutol $AUC_{0–24h}$ (for every 5 µg·h/mL)**	**0·54**	**0·33 – 0·86**	**0·00983**	**0·54**	**0·33 - 0·88**	**0·015**
**Ethambutol C_{max} (for every 1 µg/mL)**	**0·56**	**0·34 – 0·92**	**0·022**	**0·56**	**0·34 - 0·94**	**0·029**
Pyrazinamide $AUC_{0–24h}$ (for every 100 µg·h/mL)	0·87	0·64 – 1·19	0·38	0·87	0·63 - 1·19	0·37
Pyrazinamide C_{max} (for every 10 µg/mL)	0·60	0·35 – 1·02	0·060	0·58	0·34 - 1·00	0·051
**Isoniazid $AUC_{0–24h}$ (for every 5 µg·h/mL)**	**0·80**	**0·67 – 0·95**	**0·011**	**0·80**	**0·67 - 0·95**	**0·011**
**Isoniazid C_{max} (for every 1 µg/mL)**	**0·53**	**0·35 – 0·81**	**0·00295**	**0·53**	**0·35 - 0·81**	**0·00295**

**Control Regimen**	**Main Analysis Including Imputed PK**			**Sensitivity Analysis Excluding Imputed PK**		
**Predictor**	Unadjusted Hazard Ratio	Unadjusted 95% CI	Unadjusted p-value	Unadjusted Hazard Ratio	Unadjusted 95% CI	Unadjusted p-value
**PHARMACOKINETIC FACTORS**						
Rifampicin $AUC_{0–24h}$ (for every 10 µg·h/mL)	1·02	0·93 - 1·11	0·67	1·00	0·90 - 1·12	0·95
Rifampicin C_{max} (for every 1 µg/mL)	1·02	0·95 - 1·10	0·63	1·01	0·93 - 1·10	0·78
Ethambutol $AUC_{0–24h}$ (for every 5 µg·h/mL)	1·09	0·91 - 1·30	0·37	1·09	0·91 - 1·30	0·38
Ethambutol C_{max} (for every 1 µg/mL)	1·10	0·60 - 2·03	0·75	1·10	0·60 - 2·03	0·76
**Pyrazinamide $AUC_{0–24h}$ (for every 100 µg·h/mL)**	**0·38**	**0·17 - 0·82**	**0·013**	**0·44**	**0·20 - 0·99**	**0·048**
**Pyrazinamide C_{max} (for every 10 µg/mL)**	**0·35**	**0·14 - 0·90**	**0·029**	0·46	0·17 - 1·23	0·12
Isoniazid $AUC_{0–24h}$ (for every 5 µg·h/mL)	0·49	0·20 - 1·16	0·10	0·66	0·26 - 1·69	0·39
Isoniazid C_{max} (for every 1 µg/mL)	0·34	0·12 - 0·96	0·041	0·53	0·19 - 1·48	0·22

Supplementary Table 8. Sensitivity Analysis of Multivariable Cox Proportional Hazards of Tuberculosis-Related Unfavorable Outcomes by Pharmacokinetic Factors, Including and Excluding Imputed Values. Hazard ratios, confidence intervals and two-tailed p-values calculated by Cox proportional hazards regression.

**Rifapentine-Moxifloxacin Regimen**	**Main Analysis Including Imputed PK**			**Sensitivity Analysis Excluding Imputed PK**		
**Predictor**	Adjusted Hazard Ratio	Adjusted 95% CI	Adjusted p-value	Adjusted Hazard Ratio	Adjusted 95% CI	Adjusted p-value
Rifapentine AUC$_{0–24h}$ (for every 100 µg·h/mL)	0·77	0·63 - 0·95	0·015	0·76	0·61 - 0·95	0·015
Xpert MTB/RIF CT (for every 3 CT decrease)	1·43	1·07 - 1·91	0·015	1·55	1·14 - 2·10	0·00521
Extent of disease (≥50% relative to <25%/25-50%)	2·03	1·08 - 3·83	0·029	2·17	1·12 - 4·23	0·022

**Rifapentine Regimen**	**Main Analysis Including Imputed PK**			**Sensitivity Analysis Excluding Imputed PK**		
**Predictor**	Adjusted Hazard Ratio	Adjusted 95% CI	Adjusted p-value	Adjusted Hazard Ratio	Adjusted 95% CI	Adjusted p-value
Rifapentine AUC$_{0–24h}$ (for every 100 µg·h/mL)	0·65	0·54 - 0·77	0.00000053	0·65	0·54 - 0·77	0.00000071
Xpert MTB/RIF CT (for every 3 CT decrease)	1·54	1·93 - 1·24	0·00012	1·60	1·28 - 2·01	0.04
Extent of disease (≥50% relative to <25%/25-50%)	1·61	0·98 - 2·65	0·060	1·68	1·01 - 2·78	0·047
Age (for every 10-year increase)	1·37	1·13 - 1·67	0·00166	1·38	1·14 - 1·69	0·00130
Weight (for every 10 kg increase)	0·57	0·40 - 0·80	0·00124	0·55	0·38 - 0·78	0.00082

**Control Regimen**	**Main Analysis Including Imputed PK**			**Sensitivity Analysis Excluding Imputed PK**		
**Predictor**	Adjusted Hazard Ratio	Adjusted 95% CI	Adjusted p-value	Adjusted Hazard Ratio	Adjusted 95% CI	Adjusted p-value
Pyrazinamide AUC$_{0–24h}$ (for every 100 µg·h/mL)	0·36	0·15 - 0·83	0·016	0·43	0·18 - 1·03	0·06
Xpert MTB/RIF CT (for every 3 CT decrease)	1·69	1·08 - 2·63	0·021	1·95	1·16 - 3·28	0·011

A

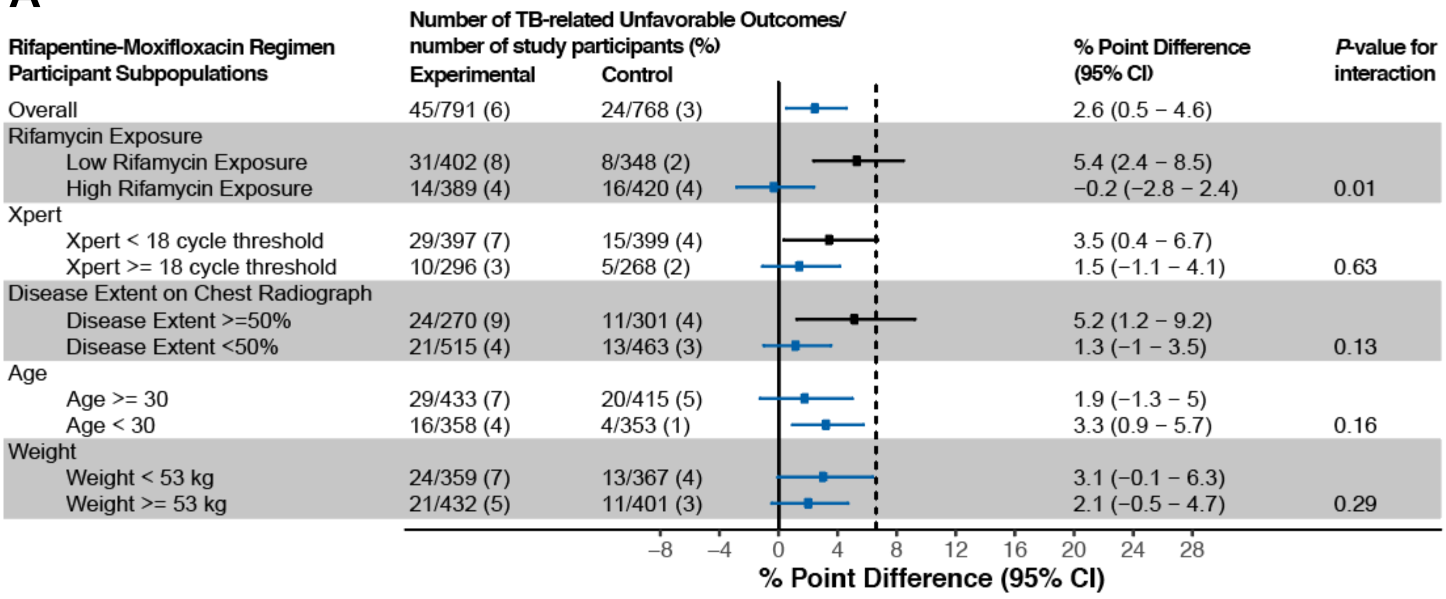

Rifapentine-Moxifloxacin Regimen Participant Subpopulations	Number of TB-related Unfavorable Outcomes/ number of study participants (%) Experimental	Control	% Point Difference (95% CI)	*P*-value for interaction
Overall	45/791 (6)	24/768 (3)	2.6 (0.5 – 4.6)	
Rifamycin Exposure				
Low Rifamycin Exposure	31/402 (8)	8/348 (2)	5.4 (2.4 – 8.5)	
High Rifamycin Exposure	14/389 (4)	16/420 (4)	−0.2 (−2.8 – 2.4)	0.01
Xpert				
Xpert < 18 cycle threshold	29/397 (7)	15/399 (4)	3.5 (0.4 – 6.7)	
Xpert >= 18 cycle threshold	10/296 (3)	5/268 (2)	1.5 (−1.1 – 4.1)	0.63
Disease Extent on Chest Radiograph				
Disease Extent >=50%	24/270 (9)	11/301 (4)	5.2 (1.2 – 9.2)	
Disease Extent <50%	21/515 (4)	13/463 (3)	1.3 (−1 – 3.5)	0.13
Age				
Age >= 30	29/433 (7)	20/415 (5)	1.9 (−1.3 – 5)	
Age < 30	16/358 (4)	4/353 (1)	3.3 (0.9 – 5.7)	0.16
Weight				
Weight < 53 kg	24/359 (7)	13/367 (4)	3.1 (−0.1 – 6.3)	
Weight >= 53 kg	21/432 (5)	11/401 (3)	2.1 (−0.5 – 4.7)	0.29

B

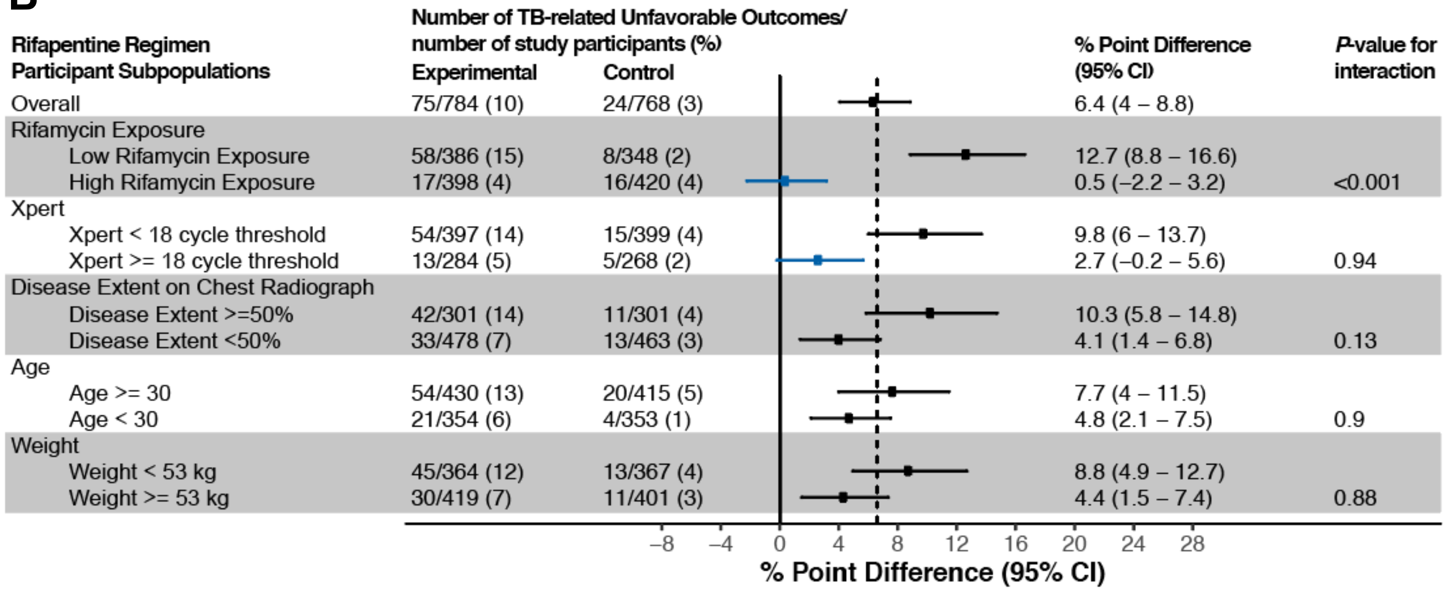

Rifapentine Regimen Participant Subpopulations	Number of TB-related Unfavorable Outcomes/ number of study participants (%) Experimental	Control	% Point Difference (95% CI)	*P*-value for interaction
Overall	75/784 (10)	24/768 (3)	6.4 (4 – 8.8)	
Rifamycin Exposure				
Low Rifamycin Exposure	58/386 (15)	8/348 (2)	12.7 (8.8 – 16.6)	
High Rifamycin Exposure	17/398 (4)	16/420 (4)	0.5 (−2.2 – 3.2)	<0.001
Xpert				
Xpert < 18 cycle threshold	54/397 (14)	15/399 (4)	9.8 (6 – 13.7)	
Xpert >= 18 cycle threshold	13/284 (5)	5/268 (2)	2.7 (−0.2 – 5.6)	0.94
Disease Extent on Chest Radiograph				
Disease Extent >=50%	42/301 (14)	11/301 (4)	10.3 (5.8 – 14.8)	
Disease Extent <50%	33/478 (7)	13/463 (3)	4.1 (1.4 – 6.8)	0.13
Age				
Age >= 30	54/430 (13)	20/415 (5)	7.7 (4 – 11.5)	
Age < 30	21/354 (6)	4/353 (1)	4.8 (2.1 – 7.5)	0.9
Weight				
Weight < 53 kg	45/364 (12)	13/367 (4)	8.8 (4.9 – 12.7)	
Weight >= 53 kg	30/419 (7)	11/401 (3)	4.4 (1.5 – 7.4)	0.88

Supplementary Figure 4. Subgroup Analyses of (A) Rifapentine-Moxifloxacin and (B) Rifapentine Regimens Stratified by Median Value of Identified Risk Factors. Two-tailed interaction p-values tested for interaction between regimen (experimental vs. control) and the covariates in a Cox proportional hazards model. For the experimental regimens, low and high rifamycin exposure based on median AUC as defined for rifapentine and for the control, median AUC for rifampicin. The vertical dotted line represents the 6·6% noninferiority margin defined in the primary analysis. Subgroups whose upper confidence interval is within the noninferiority margin are colored blue.

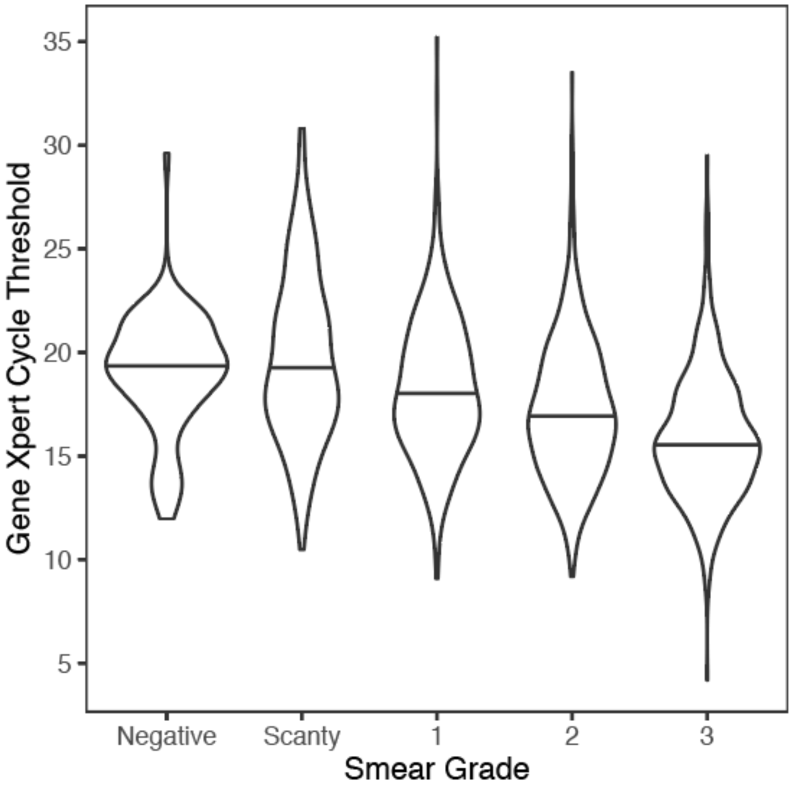

Supplementary Figure 5. Violin Plot of Xpert MTB/RIF Cycle Threshold Values by Smear Grade. The central line in each violin represents the median value. Xpert MTB/RIF cycle threshold and smear grade do not have a perfect translation, but cycle threshold decreases with increasing smear grade. Negative smear grade = 19·4 median cycle threshold, scanty smear grade = 19·1 median cycle threshold, smear grade 1+ = 18 median cycle threshold, smear grade 2+ = 16·9 median cycle threshold, smear grade 3+ = 15·5 median cycle threshold.

A

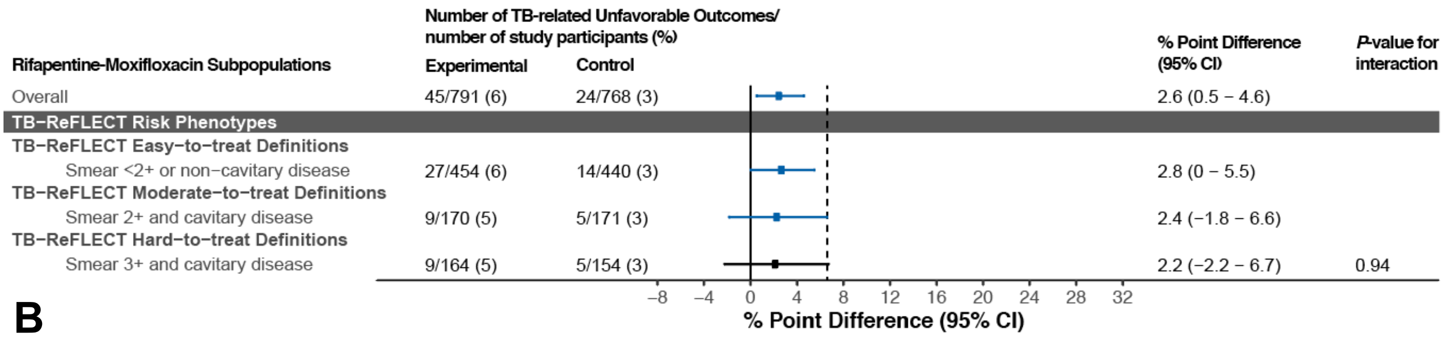

B

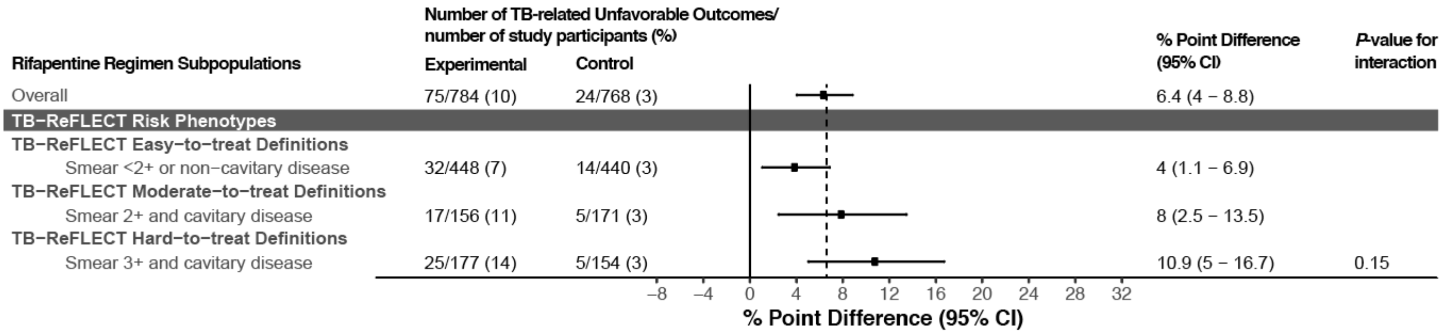

Supplementary Figure 6. Prespecified Disease Phenotype Definitions from the TB-ReFLECT Analysis are Validated in the Rifapentine Regimen. Two-tailed interaction p-values tested for interaction between regimen (experimental vs. control) and the disease phenotypes in a Cox proportional hazards model. The figure shows the results of subgroup analyses of TB-ReFLECT risk groups as defined by Imperial et al.[4] **(A)** Percentage point differences remained small across all TB-ReFLECT disease phenotypes in the rifapentine-moxifloxacin regimen. **(B)** The expected graded response is observed in the rifapentine regimen, where easier-to-treat TB had small risk differences relative to control and harder-to-treat TB had large risk differences.

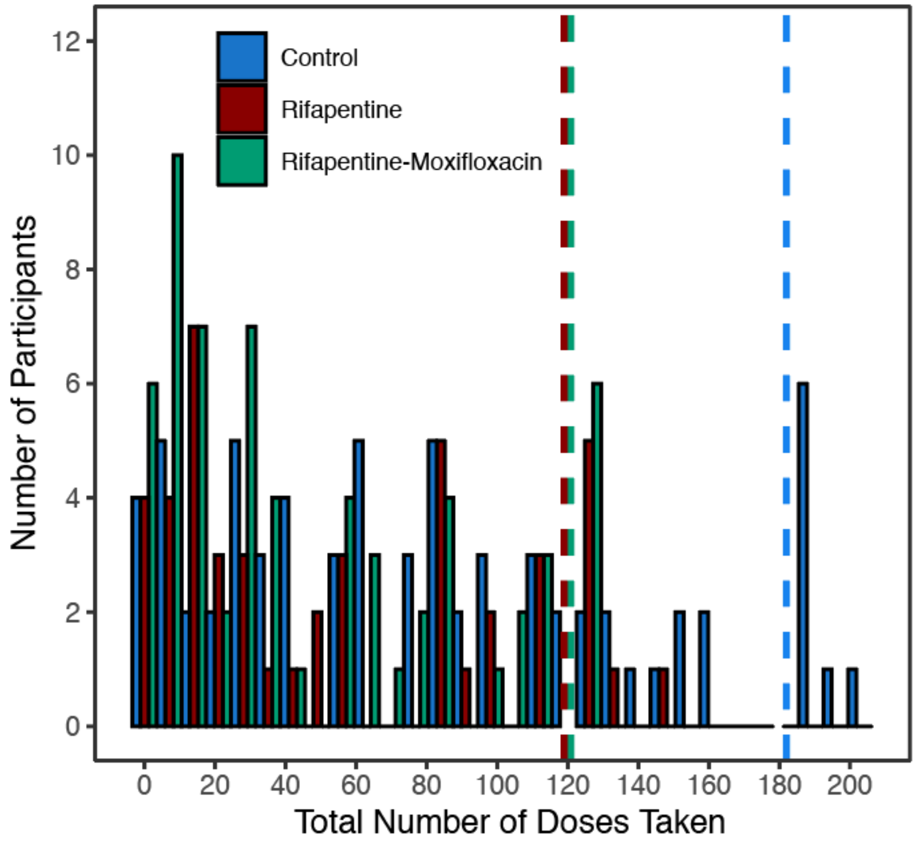

Supplementary Figure 7. Adherence Histogram of Participants in Microbiologically Eligible Population. Figure only shows participants with unexpected number of doses. The vast majority of participants completed the planned number of doses and were excluded from this figure: 182 doses for control (blue dashed line) and 119 doses for the 4-month experimental regimens (red and green dashed lines). 642/768 (83.6%) of participants in the control regimen were administered exactly 182 doses, and 636/784 (81.1%) in the rifapentine regimen and 616/791 (77.9%) in the rifapentine-moxifloxacin regimen were administered exactly 119 doses and were excluded from this figure.

Supplementary Table 9. Adherence Unadjusted and Adjusted Hazard Ratios for Tuberculosis-Related Unfavorable Outcomes Among Participants. Analysis performed in the microbiologically eligible population. In adjusted analyses, hazard ratios are adjusted for significant baseline and PK factors identified in Figure 1 (also listed in the table). Hazard ratios, confidence intervals and two-tailed p-values calculated by Cox proportional hazards regression.

Rifapentine-Moxifloxacin Regimen

**Predictor**	Unadjusted Hazard Ratio	Unadjusted 95% CI	Unadjusted p-value	Adjusted Hazard Ratio	Adjusted 95% CI	Adjusted p-value
Rifapentine AUC$_{0–24h}$ (for every 100 µg·h/mL)	0·77	0·64 - 0·93	0·00702	0·74	0·60 - 0·91	0·00420
Xpert MTB/RIF CT (for every 3 CT decrease)	1·48	1·98 - 1·10	0·00904	1·47	1·96 - 1·10	0·00973
Extent of disease (≥50% relative to <25%/25-50%)	2·19	1·22 - 3·94	0·00876	2·38	1·22 - 4·62	0·011
Adherence (for every week of missed doses)	1·22	1·11 - 1·33	<0·001	1·31	1·19 – 1·44	<0·001

Rifapentine Regimen

**Predictor**	Unadjusted Hazard Ratio	Unadjusted 95% CI	Unadjusted p-value	Adjusted Hazard Ratio	Adjusted 95% CI	Adjusted p-value
Rifapentine AUC$_{0–24h}$ (for every 100 µg·h/mL)	0·65	0·55 - 0·76	0.00000018	0·64	0·54 - 0·76	0.0000004
Xpert MTB/RIF CT (for every 3 CT decrease)	1·63	2·02 - 1·32	0.0000062	1·54	1·93 - 1·24	0.00013
Extent of disease (≥50% relative to <25%/25-50%)	2·10	1·33 - 3·32	0·00139	1·68	1·02 - 2·78	0·042
Age (for every 10-year increase)	1·45	1·23 - 1·72	0.000013	1·37	1·12 - 1·67	0·00192
Weight (for every 10 kg increase)	0·61	0·44 - 0·83	0·00152	0·57	0·40 - 0·80	0·00139
Adherence (for every week of missed doses)	1·16	1·05 – 1·28	0·00456	1·10	0·96 - 1·27	0·17

Control Regimen

**Predictor**	Unadjusted Hazard Ratio	Unadjusted 95% CI	Unadjusted p-value	Adjusted Hazard Ratio	Adjusted 95% CI	Adjusted p-value
Pyrazinamide AUC$_{0–24h}$ (for every 100 µg·h/mL)	0·37	0·17 - 0·80	0·011	0·38	0·16 - 0·88	0·024
Xpert MTB/RIF CT (for every 3 CT decrease)	1·65	2·57 - 1·06	0·025	1·77	2·75 - 1·14	0·010
Adherence (for every week of missed doses)	1·45	1·23 – 1·72	0.00000001	1·37	1·12 – 1·67	0.0000003

Supplementary Table 10. Medical Dictionary for Regulatory Activities (MedDRA) Coded Grade 3 or Higher Adverse Events by Regimen. The number and percent of participants are reported in the first column of each regimen, and the number of events is reported in the second column.

SYSTEM ORGAN CLASS	PREFERRED TERM	CONTROL REGIMEN N=825		RIFAPENTINE N=835		RIFAPENTINE-MOXIFLOXACIN N=846	
		N (%)	N events	N (%)	N events	N (%)	N events
BLOOD AND LYMPHATIC SYSTEM DISORDERS	ANAEMIA	5 (0·61)	5	1 (0·12)	1	5 (0·59)	5
	HAEMOLYTIC ANAEMIA	1 (0·12)	1	0 (0)	0	0 (0)	0
	HYPOCHROMIC ANAEMIA	1 (0·12)	1	0 (0)	0	0 (0)	0
	IRON DEFICIENCY ANAEMIA	1 (0·12)	1	0 (0)	0	0 (0)	0
	LEUKOCYTOSIS	0 (0)	0	0 (0)	0	1 (0·12)	1
	LEUKOPENIA	0 (0)	0	3 (0·36)	3	1 (0·12)	1
	LYMPHOPENIA	0 (0)	0	0 (0)	0	3 (0·35)	3
	MICROCYTIC ANAEMIA	1 (0·12)	1	0 (0)	0	0 (0)	0
	NEUTROPENIA	47 (5·7)	53	33 (3·95)	36	56 (6·62)	67
	THROMBOCYTOPENIA	0 (0)	0	1 (0·12)	1	1 (0·12)	1
	THROMBOTIC THROMBOCYTOPENIC PURPURA	0 (0)	0	0 (0)	0	1 (0·12)	1
HEPATOBILIARY DISORDERS	CHOLELITHIASIS	0 (0)	0	1 (0·12)	1	0 (0)	0
	GAMMA GT RAISED	0 (0)	0	0 (0)	0	1 (0·12)	1
	HEPATITIS	26 (3·15)	27	25 (2·99)	28	38 (4·49)	40
	HYPERBILIRUBINAEMIA	0 (0)	0	1 (0·12)	1	2 (0·24)	2
VASCULAR DISORDERS	AORTIC ANEURYSM	0 (0)	0	0 (0)	0	1 (0·12)	1
	AORTIC THROMBOSIS	1 (0·12)	1	0 (0)	0	0 (0)	0
	DEEP VEIN THROMBOSIS	3 (0·36)	3	1 (0·12)	1	1 (0·12)	1
	HYPERTENSION	16 (1·94)	18	16 (1·92)	18	13 (1·54)	18
INFECTIONS AND INFESTATIONS	BODY TINEA	0 (0)	0	0 (0)	0	1 (0·12)	1
	BONE TUBERCULOSIS	1 (0·12)	1	0 (0)	0	0 (0)	0
	CONJUNCTIVITIS	0 (0)	0	0 (0)	0	1 (0·12)	1
	CONJUNCTIVITIS VIRAL	1 (0·12)	1	0 (0)	0	0 (0)	0
	DENGUE FEVER	0 (0)	0	1 (0·12)	1	0 (0)	0
	DISSEMINATED TUBERCULOSIS	0 (0)	0	1 (0·12)	1	0 (0)	0
	EXTRAPULMONARY TUBERCULOSIS	0 (0)	0	1 (0·12)	1	0 (0)	0
	GASTROENTERITIS	1 (0·12)	1	0 (0)	0	0 (0)	0
	HEPATITIS A	0 (0)	0	1 (0·12)	1	0 (0)	0
	HEPATITIS C	0 (0)	0	0 (0)	0	1 (0·12)	1
	HIV INFECTION CDC GROUP IV SUBGROUP C1	0 (0)	0	1 (0·12)	1	0 (0)	0
	LOWER RESPIRATORY TRACT INFECTION	0 (0)	0	1 (0·12)	1	0 (0)	0
	LUNG ABSCESS	0 (0)	0	1 (0·12)	1	0 (0)	0
	MALARIA	3 (0·36)	5	0 (0)	0	2 (0·24)	2
	OOPHORITIS	0 (0)	0	1 (0·12)	1	0 (0)	0
	ORCHITIS	0 (0)	0	1 (0·12)	1	0 (0)	0
	PARACOCCIDIOIDES INFECTION	1 (0·12)	1	0 (0)	0	0 (0)	0
	PELVIC INFLAMMATORY DISEASE	2 (0·24)	2	0 (0)	0	0 (0)	0
	PERICARDITIS TUBERCULOUS	0 (0)	0	0 (0)	0	1 (0·12)	1
	PNEUMOCYSTIS JIROVECII PNEUMONIA	1 (0·12)	1	0 (0)	0	0 (0)	0
	PNEUMONIA	2 (0·24)	2	1 (0·12)	1	4 (0·47)	5
	PNEUMONIA BACTERIAL	2 (0·24)	2	2 (0·24)	2	1 (0·12)	1
	PULMONARY TUBERCULOSIS	0 (0)	0	2 (0·24)	2	2 (0·24)	2
	RESPIRATORY TRACT INFECTION	1 (0·12)	1	0 (0)	0	0 (0)	0
	SEPSIS	1 (0·12)	1	0 (0)	0	0 (0)	0
	TUBERCULOSIS	1 (0·12)	1	0 (0)	0	0 (0)	0
	URINARY TRACT INFECTION	1 (0·12)	1	1 (0·12)	1	0 (0)	0
	VULVOVAGINAL CANDIDIASIS	0 (0)	0	0 (0)	0	1 (0·12)	1
PREGNANCY, PUERPERIUM AND PERINATAL CONDITIONS	ABORTION SPONTANEOUS	0 (0)	0	0 (0)	0	1 (0·12)	1
	COMPLICATION OF PREGNANCY	0 (0)	0	0 (0)	0	1 (0·12)	1
	PRE-ECLAMPSIA	1 (0·12)	2	0 (0)	0	0 (0)	0

	PREGNANCY	20 (2·42)	22	9 (1·08)	10	10 (1·18)	11
	PRETERM PREMATURE RUPTURE OF MEMBRANES	0 (0)	0	0 (0)	0	1 (0·12)	1
METABOLISM AND NUTRITION DISORDERS	ABNORMAL LOSS OF WEIGHT	0 (0)	0	0 (0)	0	1 (0·12)	1
	ABNORMAL WEIGHT GAIN	0 (0)	0	1 (0·12)	1	0 (0)	0
	DIABETES MELLITUS	1 (0·12)	1	0 (0)	0	0 (0)	0
	DIABETES MELLITUS INADEQUATE CONTROL	3 (0·36)	5	2 (0·24)	2	3 (0·35)	6
	DIABETIC KETOACIDOSIS	0 (0)	0	0 (0)	0	1 (0·12)	2
	GOUT	1 (0·12)	1	0 (0)	0	0 (0)	0
	HYPERGLYCAEMIA	1 (0·12)	1	1 (0·12)	1	1 (0·12)	1
	HYPERKALAEMIA	4 (0·48)	4	0 (0)	0	2 (0·24)	2
	HYPOALBUMINAEMIA	0 (0)	0	2 (0·24)	2	0 (0)	0
	HYPOGLYCAEMIA	1 (0·12)	1	0 (0)	0	0 (0)	0
	HYPONATRAEMIA	0 (0)	0	1 (0·12)	2	1 (0·12)	1
	PSEUDOHYPERKALAEMIA	1 (0·12)	1	0 (0)	0	1 (0·12)	1
	TYPE 2 DIABETES MELLITUS	0 (0)	0	0 (0)	0	1 (0·12)	1
RESPIRATORY, THORACIC AND MEDIASTINAL DISORDERS	BRONCHIECTASIS	0 (0)	0	0 (0)	0	1 (0·12)	1
	BRONCHOSPASM	0 (0)	0	0 (0)	0	1 (0·12)	1
	CHRONIC OBSTRUCTIVE PULMONARY DISEASE	1 (0·12)	2	0 (0)	0	0 (0)	0
	DYSPNOEA	0 (0)	0	2 (0·24)	2	0 (0)	0
	HAEMOPTYSIS	5 (0·61)	6	3 (0·36)	3	3 (0·35)	3
	PLEURAL EFFUSION	0 (0)	0	0 (0)	0	1 (0·12)	1
	PNEUMOTHORAX	0 (0)	0	0 (0)	0	1 (0·12)	1
	PULMONARY EMBOLISM	3 (0·36)	3	1 (0·12)	1	0 (0)	0
INJURY, POISONING AND PROCEDURAL COMPLICATIONS	ALCOHOL POISONING	0 (0)	0	1 (0·12)	1	0 (0)	0
	CRANIOCEREBRAL INJURY	0 (0)	0	1 (0·12)	1	0 (0)	0
	DOCUMENTED HYPERSENSITIVITY TO ADMINISTERED PRODUCT	1 (0·12)	1	0 (0)	0	0 (0)	0
	EYE INJURY	1 (0·12)	1	0 (0)	0	0 (0)	0
	HAND FRACTURE	1 (0·12)	1	0 (0)	0	0 (0)	0
	HUMERUS FRACTURE	1 (0·12)	1	0 (0)	0	0 (0)	0
	INJURY	0 (0)	0	0 (0)	0	1 (0·12)	1
	LIMB INJURY	1 (0·12)	1	0 (0)	0	0 (0)	0
	OVERDOSE	1 (0·12)	1	1 (0·12)	1	0 (0)	0
	ROAD TRAFFIC ACCIDENT	0 (0)	0	1 (0·12)	1	0 (0)	0
	STAB WOUND	0 (0)	0	2 (0·24)	2	0 (0)	0
	THERMAL BURN	1 (0·12)	1	0 (0)	0	0 (0)	0
	TIBIA FRACTURE	1 (0·12)	1	0 (0)	0	0 (0)	0
	ULNA FRACTURE	1 (0·12)	1	0 (0)	0	0 (0)	0
SKIN AND SUBCUTANEOUS TISSUE DISORDERS	ANGIOEDEMA	0 (0)	0	1 (0·12)	1	0 (0)	0
	DRUG REACTION WITH EOSINOPHILIA AND SYSTEMIC SYMPTOMS	1 (0·12)	1	0 (0)	0	0 (0)	0
	PRURITUS	0 (0)	0	1 (0·12)	1	1 (0·12)	1
	RASH GENERALISED	0 (0)	0	1 (0·12)	1	1 (0·12)	1
	RASH MACULO-PAPULAR	0 (0)	0	0 (0)	0	1 (0·12)	1
	RASH PRURITIC	0 (0)	0	2 (0·24)	2	0 (0)	0
	URTICARIA	0 (0)	0	1 (0·12)	1	4 (0·47)	4
NERVOUS SYSTEM DISORDERS	CENTRAL NERVOUS SYSTEM LESION	1 (0·12)	2	0 (0)	0	0 (0)	0
	CEREBRAL INFARCTION	0 (0)	0	0 (0)	0	1 (0·12)	1
	CEREBROVASCULAR ACCIDENT	0 (0)	0	0 (0)	0	1 (0·12)	1
	EPILEPSY	1 (0·12)	1	0 (0)	0	0 (0)	0
	GUILLAIN-BARRE SYNDROME	0 (0)	0	0 (0)	0	1 (0·12)	1
	NEUROPATHY PERIPHERAL	0 (0)	0	1 (0·12)	1	0 (0)	0
	SEIZURE	1 (0·12)	1	0 (0)	0	0 (0)	0
	SYNCOPE	0 (0)	0	0 (0)	0	2 (0·24)	2

	TEMPORAL LOBE EPILEPSY	1 (0·12)	1	0 (0)	0	0 (0)	0
EYE DISORDERS	ASTIGMATISM	0 (0)	0	0 (0)	0	1 (0·12)	1
	BLEPHARITIS	0 (0)	0	1 (0·12)	1	0 (0)	0
	CATARACT	0 (0)	0	0 (0)	0	1 (0·12)	1
	CONJUNCTIVITIS ALLERGIC	0 (0)	0	0 (0)	0	1 (0·12)	1
	DIABETIC RETINOPATHY	1 (0·12)	1	0 (0)	0	0 (0)	0
	OPTIC NEUROPATHY	1 (0·12)	1	0 (0)	0	0 (0)	0
	REFRACTION DISORDER	0 (0)	0	0 (0)	0	1 (0·12)	1
	VISUAL ACUITY REDUCED	2 (0·24)	2	0 (0)	0	0 (0)	0
	VITRITIS	0 (0)	0	0 (0)	0	1 (0·12)	1
INVESTIGATIONS	BLOOD BILIRUBIN INCREASED	0 (0)	0	0 (0)	0	1 (0·12)	1
	BLOOD PRESSURE INCREASED	1 (0·12)	1	2 (0·24)	2	2 (0·24)	2
	PREGNANCY TEST FALSE POSITIVE	1 (0·12)	1	1 (0·12)	1	1 (0·12)	1
	WEIGHT DECREASED	1 (0·12)	1	0 (0)	0	0 (0)	0
NEOPLASMS BENIGN, MALIGNANT AND UNSPECIFIED (INCL CYSTS AND POLYPS)	ANOGENITAL WARTS	1 (0·12)	1	0 (0)	0	0 (0)	0
	BLADDER TRANSITIONAL CELL CARCINOMA	0 (0)	0	1 (0·12)	1	0 (0)	0
	BREAST CANCER	1 (0·12)	1	0 (0)	0	0 (0)	0
	LYMPHOMA	0 (0)	0	1 (0·12)	1	0 (0)	0
	NEOPLASM MALIGNANT	1 (0·12)	1	0 (0)	0	0 (0)	0
	OESOPHAGEAL CARCINOMA	0 (0)	0	0 (0)	0	1 (0·12)	1
	PAPILLARY THYROID CANCER	1 (0·12)	1	0 (0)	0	0 (0)	0
	PERIPHERAL NERVE SHEATH TUMOUR MALIGNANT	1 (0·12)	1	0 (0)	0	0 (0)	0
	SQUAMOUS CELL CARCINOMA	1 (0·12)	1	0 (0)	0	0 (0)	0
	SQUAMOUS CELL CARCINOMA OF THE TONGUE	1 (0·12)	1	0 (0)	0	0 (0)	0
GASTROINTESTINAL DISORDERS	GASTRITIS	0 (0)	0	0 (0)	0	1 (0·12)	1
	PANCREATITIS ACUTE	1 (0·12)	1	0 (0)	0	0 (0)	0
	PEPTIC ULCER	0 (0)	0	0 (0)	0	1 (0·12)	1
	PNEUMATOSIS INTESTINALIS	1 (0·12)	2	0 (0)	0	0 (0)	0
	SMALL INTESTINAL OBSTRUCTION	1 (0·12)	1	0 (0)	0	0 (0)	0
	VOMITING	0 (0)	0	1 (0·12)	1	0 (0)	0
GENERAL DISORDERS AND ADMINISTRATION SITE CONDITIONS	ADVERSE DRUG REACTION	0 (0)	0	0 (0)	0	2 (0·24)	3
	DEATH	1 (0·12)	1	1 (0·12)	1	0 (0)	0
	DRUG INTOLERANCE	1 (0·12)	1	0 (0)	0	0 (0)	0
	PYREXIA	1 (0·12)	1	0 (0)	0	0 (0)	0
MUSCULOSKELETAL AND CONNECTIVE TISSUE DISORDERS	ARTHRALGIA	2 (0·24)	2	0 (0)	0	1 (0·12)	1
	COSTOCHONDRITIS	1 (0·12)	1	0 (0)	0	0 (0)	0
	INTERVERTEBRAL DISC DISORDER	0 (0)	0	1 (0·12)	1	0 (0)	0
	SACROILIITIS	1 (0·12)	1	0 (0)	0	0 (0)	0
	SPINAL OSTEOARTHRITIS	1 (0·12)	1	0 (0)	0	0 (0)	0
CARDIAC DISORDERS	CARDIAC FAILURE CONGESTIVE	0 (0)	0	0 (0)	0	1 (0·12)	2
	COR PULMONALE	1 (0·12)	1	0 (0)	0	0 (0)	0
	LONG QT SYNDROME	0 (0)	0	0 (0)	0	1 (0·12)	1
	RIGHT VENTRICULAR FAILURE	0 (0)	0	0 (0)	0	1 (0·12)	1
PSYCHIATRIC DISORDERS	BRIEF PSYCHOTIC DISORDER, WITH POSTPARTUM ONSET	1 (0·12)	1	0 (0)	0	0 (0)	0
	DISORIENTATION	0 (0)	0	0 (0)	0	1 (0·12)	1
	SUICIDE ATTEMPT	0 (0)	0	1 (0·12)	1	1 (0·12)	1
IMMUNE SYSTEM DISORDERS	DRUG HYPERSENSITIVITY	0 (0)	0	0 (0)	0	2 (0·24)	2
RENAL AND URINARY DISORDERS	RENAL IMPAIRMENT	0 (0)	0	0 (0)	0	1 (0·12)	1
	RENAL TUBULAR NECROSIS	0 (0)	0	0 (0)	0	1 (0·12)	1
CONGENITAL, FAMILIAL AND GENETIC DISORDERS	CONGENITAL ANOMALY	0 (0)	0	1 (0·12)	1	0 (0)	0

Supplementary Table 11. Univariate and Multivariable Safety Analysis of Any Grade 3 or Higher Adverse Events in Participants Receiving Rifapentine-Moxifloxacin Regimen in the Safety Population. Baseline clinical factors and individual drug pharmacokinetic estimates were evaluated in Univariate and multivariable logistic regression models as potential risk factors for the occurrence of any grade 3 or higher adverse events. Odds ratios, confidence intervals and two-tailed p-values calculated by logistic regression.

Predictor	Unadjusted Odds Ratio	Unadjusted 95% CI	Unadjusted p-value	Adjusted Odds Ratio	Adjusted 95% CI	Adjusted p-value
DEMOGRAPHIC FACTORS						
**Age (for every 10 years)**	**1·23**	**1·07 - 1·42**	**0·00415**	**1·22**	**1·06 - 1·41**	**0·0058**
Female sex (relative to male)	1·14	0·78 - 1·64	0·49			
WT (for every 10 kg)	0·94	0·77 - 1·12	0·49			
BMI (for every 5 units)	1·08	0·83 - 1·37	0·57			
Asian Race (relative to Black)	1·27	0·75 - 2·07	0·36			
Mixed Race (relative to Black)	0·76	0·44 - 1·27	0·31			
African clinical site (relative to non-African)	1·02	0·70 - 1·52	0·91			
BASELINE CLINICAL FACTORS						
Xpert MTB/RIF CT (for every 3 CT increase)	0·95	0·81 - 1·11	0·51			
Time to Detection on Sputum Liquid Culture (for every 5 day increase)	0·96	0·77 - 1·17	0·68			
Presence of Cavitation	0·90	0·62 - 1·33	0·60			
Aggregate cavity size <4cm (relative to no cavities)	0·82	0·53 - 1·28	0·39			
Aggregate cavity size ≥4cm (relative to no cavities)	0·80	0·52 - 1·24	0·32			
Extent of disease (<25% relative to 25-50%)	1·06	0·67 - 1·73	0·81			
Extent of disease (≥50% relative to 25-50%)	1·22	0·75 - 2·03	0·42			
Smear grade 0 relative to 2	0·78	0·32 - 2·11	0·60			
Smear grade 0·5 relative to 2	0·76	0·32 - 2·05	0·57			
Smear grade 1 relative to 2	0·89	0·38 - 2·33	0·79			
Smear grade 3 relative to 2	0·70	0·30 - 1·87	0·45			
**Karnofsky score (for every 10)**	**1·32**	**1·01 - 1·75**	**0·05**			
Living with HIV (relative to without HIV)	0·68	0·32 - 1·29	0·27			
History of Diabetes (relative to no history)	1·78	0·80 - 3·67	0·14			
**History of liver disease**	**8·84**	**1·71 - 64·2**	**0·012**	**7·43**	**1·42 - 54·3**	**0·022**
PHARMACOKINETIC FACTORS						
Rifapentine AUC_{0-24h} (for every 100 µg·h/mL)	1·02	0·92 - 1·13	0·70			
Rifapentine C_{max} (for every 10 µg/mL**)**	1·00	0·81 - 1·24	0·98			
Moxifloxacin AUC_{0-24h} (for every 5 µg·h/mL)	1·03	0·91 - 1·16	0·59			
Moxifloxacin C_{max} (for every 1 µg/mL)	1·09	0·85 - 1·38	0·47			
**Pyrazinamide AUC_{0-24h} (for every 100 µg·h/mL)**	**1·22**	**1·02 - 1·45**	**0·03**	**1·23**	**1·03 - 1·47**	**0·022**
Pyrazinamide C_{max} (for every 10 µg/mL)	1·17	0·93 - 1·46	0·17			
Isoniazid AUC_{0-24h} (for every 5 µg·h/mL)	1·08	0·97 - 1·19	0·17			
Isoniazid C_{max} (for every 1 µg/mL)	1·10	0·87 - 1·36	0·42			

Supplementary Table 12. Univariate and Multivariable Safety Analysis of Any Grade 3 or Higher Adverse Events in Participants Receiving Control Regimen in the Safety Population. Baseline clinical factors and individual drug pharmacokinetic estimates were evaluated in Univariate and multivariable logistic regression models as potential risk factors for the occurrence of any grade 3 or higher adverse events. Odds ratios, confidence intervals and two-tailed p-values calculated by logistic regression.

Predictor	Unadjusted Odds Ratio	Unadjusted 95% CI	Unadjusted p-value	Adjusted Odds Ratio	Adjusted 95% CI	Adjusted p-value
DEMOGRAPHIC FACTORS						
Age (for every 10 years)	1·05	0·90 - 1·21	0·54			
**Female sex (relative to male)**	**1·56**	**1·09 - 2·22**	**0·016**	**1·74**	**1·17 - 2·56**	**0·00519**
WT (for every 10 kg)	1·13	0·93 - 1·36	0·21			
**BMI (for every 5 units)**	**1·30**	**1·01 - 1·68**	**0·04**			
Asian Race (relative to Black)	0·97	0·55 - 1·64	0·92			
Mixed Race (relative to Black)	0·60	0·32 - 1·06	0·093			
African clinical site (relative to non-African)	0·85	0·58 - 1·25	0·40			
BASELINE CLINICAL FACTORS						
**Xpert MTB/RIF CT (for every 3 CT increase)**	**1·23**	**1·06 - 1·43**	**0·0049**	**1·22**	**1·05 - 1·42**	**0·00875**
Time to Detection on Sputum Liquid Culture (for every 5 day increase)	1·09	0·88 - 1·33	0·39			
Presence of Cavitation	0·82	0·56 - 1·20	0·30			
Aggregate cavity size <4cm (relative to no cavities)	0·87	0·56 - 1·34	0·52			
Aggregate cavity size ≥4cm (relative to no cavities)	0·72	0·47 - 1·10	0·13			
Extent of disease (<25% relative to 25-50%)	1·10	0·66 - 1·88	0·73			
Extent of disease (≥50% relative to 25-50%)	1·31	0·79 - 2·25	0·31			
Smear grade 0 relative to 2	1·26	0·47 - 4·03	0·66			
Smear grade 0·5 relative to 2	0·94	0·35 - 2·95	0·90			
Smear grade 1 relative to 2	1·08	0·42 - 3·36	0·88			
Smear grade 3 relative to 2	0·83	0·31 - 2·61	0·72			
Karnofsky score (for every 10)	0·81	0·63 - 1·05	0·11			
Living with HIV (relative to without HIV)	1·16	0·61 - 2·05	0·64			
**History of Diabetes (relative to no history)**	**2·53**	**1·14 - 5·35**	**0·017**			
History of liver disease	0·84	0·04 - 5·24	0·87			
PHARMACOKINETIC FACTORS						
Rifampicin AUC_{0-24h} (for every 10 µg·h/mL)	1·03	0·98 - 1·07	0·26			
Rifampicin C_{max} (for every 1 µg/mL)	1·03	0·99 - 1·06	0·12			
**Ethambutol AUC_{0-24h} (for every 5 µg·h/mL)**	**1·40**	**1·01 - 1·94**	**0·045**			
Ethambutol C_{max} (for every 1 µg/mL)	1·40	0·99 - 1·94	0·051			
Pyrazinamide AUC_{0-24h} (for every 100 µg·h/mL)	1·16	0·94 - 1·42	0·15			
**Pyrazinamide C_{max} (for every 10 µg/mL)**	**1·52**	**1·11 - 2·06**	**0·00792**			
Isoniazid AUC_{0-24h} (for every 5 µg·h/mL)	1·02	0·94 - 1·11	0·62			
Isoniazid C_{max} (for every 1 µg/mL)	1·17	0·90 - 1·52	0·24			

Supplementary Table 13. Univariate and Multivariable Safety Analysis of Any Grade 3 or Higher Adverse Events in Participants Receiving Rifapentine Regimen in the Safety Population. Baseline clinical factors and individual drug pharmacokinetic estimates were evaluated in Univariate and multivariable logistic regression models as potential risk factors for the occurrence of any grade 3 or higher adverse events. Odds ratios, confidence intervals and two-tailed p-values calculated by logistic regression.

Predictor	Unadjusted Odds Ratio	Unadjusted 95% CI	Unadjusted p-value	Adjusted Odds Ratio	Adjusted 95% CI	Adjusted p-value
DEMOGRAPHIC FACTORS						
**Age (for every 10 years)**	**1·20**	**1·03 - 1·39**	**0·020**			
Female sex (relative to male)	1·39	0·92 - 2·08	0·12			
WT (for every 10 kg)	0·91	0·72 - 1·14	0·44			
BMI (for every 5 units)	1·18	0·88 - 1·55	0·25			
**Asian Race (relative to Black)**	**2·59**	**1·55 - 4·24**	**0·00021**	**2·44**	**1·45 - 3·99**	**<0·001**
Mixed Race (relative to Black)	0·91	0·48 - 1·62	0·76			
**African clinical site (relative to non-African)**	**0·64**	**0·43 - 0·97**	**0·033**			
BASELINE CLINICAL FACTORS						
Xpert MTB/RIF CT (for every 3 CT increase)	1·11	0·93 - 1·32	0·22			
Time to Detection on Sputum Liquid Culture (for every 5 day increase)	1·09	0·87 - 1·33	0·43			
Presence of Cavitation	0·86	0·57 - 1·33	0·50			
Aggregate cavity size <4cm (relative to no cavities)	1·06	0·66 - 1·73	0·80			
Aggregate cavity size ≥4cm (relative to no cavities)	0·66	0·41 - 1·08	0·099			
Extent of disease (25-50% relative to <25%)	1·84	1·07 - 3·34	0·035			
Extent of disease (>50% relative to <25%)	0·87	0·47 - 1·66	0·67			
Smear grade 0 relative to 2	1·60	0·56 - 5·76	0·41			
Smear grade 0·5 relative to 2	1·69	0·62 - 5·94	0·35			
Smear grade 1 relative to 2	1·74	0·65 - 6·05	0·32			
Smear grade 3 relative to 2	1·11	0·40 - 3·96	0·85			
Karnofsky score (for every 10)	1·25	0·92 - 1·72	0·17			
Living with HIV (relative to without HIV)	1·25	0·62 - 2·32	0·51			
History of Diabetes (relative to no history)	2·81	0·87 - 7·88	0·06			
**History of liver disease**	**8·27**	**1·80 - 42·4**	**0·00611**	**5·74**	**1·19 - 30·5**	**0·027**
PHARMACOKINETIC FACTORS						
Rifapentine AUC_{0-24h} (for every 100 µg·h/mL)	1·03	0·92 - 1·15	0·59			
Rifapentine C_{max} (for every 10 µg/mL)	1·06	0·83 - 1·34	0·62			
Ethambutol AUC_{0-24h} (for every 5 µg·h/mL)	1·33	0·99 - 1·81	0·057			
Ethambutol C_{max} (for every 1 µg/mL)	1·26	0·95 - 1·67	0·099			
Pyrazinamide AUC_{0-24h} (for every 100 µg·h/mL)	0·96	0·74 - 1·22	0·75			
Pyrazinamide C_{max} (for every 10 µg/mL)	1·19	0·82 - 1·70	0·34			
Isoniazid AUC_{0-24h} (for every 5 µg·h/mL)	0·99	0·87 - 1·11	0·81			
Isoniazid C_{max} (for every 1 µg/mL)	1·12	0·83 - 1·50	0·43			

Supplementary Table 14. Univariate Logistic Regression Safety Sensitivity Analysis of Pharmacokinetic Factors Including and Excluding Imputed Values. (A) Rifapentine-Moxifloxacin Regimen, (B) Rifapentine Regimen, (C) Control Regimen. Odds ratios, confidence intervals and two-tailed p-values calculated by logistic regression.

**Rifapentine-Moxifloxacin Regimen**	**Main Analysis Including Imputed PK**			**Sensitivity Analysis Excluding Imputed PK**		
**Predictor**	Unadjusted Odds Ratio	Unadjusted 95% CI	Unadjusted p-value	Unadjusted Odds Ratio	Unadjusted 95% CI	Unadjusted p-value
**PHARMACOKINETIC FACTORS**						
Rifapentine $AUC_{0–24h}$ (for every 100 µg·h/mL)	1·02	0·92 - 1·13	0·70	1·02	0·92 - 1·12	0·77
Rifapentine C_{max} (for every 10 µg/mL)	1·00	0·81 - 1·24	0·98	1·02	0·81 - 1·26	0·88
Moxifloxacin $AUC_{0–24h}$ (for every 5 µg·h/mL)	1·03	0·91 - 1·16	0·59	1·04	0·91 - 1·17	0·55
Moxifloxacin C_{max} (for every 1 µg/mL)	1·09	0·85 - 1·38	0·47	1·06	0·82 - 1·36	0·63
**Pyrazinamide $AUC_{0–24h}$ (for every 100 µg·h/mL)**	**1·22**	**1·02 - 1·45**	**0·03**	**1·23**	**1·02 - 1·48**	**0·026**
Pyrazinamide C_{max} (for every 10 µg/mL)	1·17	0·93 - 1·46	0·17	1·13	0·89 - 1·42	0·29
Isoniazid $AUC_{0–24h}$ (for every 5 µg·h/mL)	1·08	0·97 - 1·19	0·17	1·10	0·98 - 1·22	0·17
Isoniazid C_{max} (for every 1 µg/mL)	1·10	0·87 - 1·36	0·42	1·07	0·85 - 1·34	0·42

**Rifapentine Regimen**	**Main Analysis Including Imputed PK**			**Sensitivity Analysis Excluding Imputed PK**		
**Predictor**	Unadjusted Odds Ratio	Unadjusted 95% CI	Unadjusted p-value	Unadjusted Odds Ratio	Unadjusted 95% CI	Unadjusted p-value
**PHARMACOKINETIC FACTORS**						
Rifapentine $AUC_{0–24h}$ (for every 100 µg·h/mL)	1·03	0·92 - 1·15	0·59	1·03	0·92 - 1·15	0·62
Rifapentine C_{max} (for every 10 µg/mL)	1·06	0·83 - 1·34	0·62	1·07	0·84 - 1·36	0·57
**Ethambutol $AUC_{0–24h}$ (for every 5 µg·h/mL)**	1·33	0·99 - 1·81	0·057	**1·40**	**1·02 - 1·96**	**0·037**
Ethambutol C_{max} (for every 1 µg/mL)	1·26	0·95 - 1·67	0·099	1·31	0·98 - 1·75	0·061
Pyrazinamide $AUC_{0–24h}$ (for every 100 µg·h/mL)	0·96	0·74 - 1·22	0·75	0·99	0·76 - 1·25	0·93
Pyrazinamide C_{max} (for every 10 µg/mL)	1·19	0·82 - 1·70	0·34	1·26	0·86 - 1·81	0·21
Isoniazid $AUC_{0–24h}$ (for every 5 µg·h/mL)	0·99	0·87 - 1·11	0·81	0·99	0·87 - 1·12	0·81
Isoniazid C_{max} (for every 1 µg/mL)	1·12	0·83 - 1·50	0·43	1·11	0·82 - 1·48	0·43

**Control Regimen**	**Main Analysis Including Imputed PK**			**Sensitivity Analysis Excluding Imputed PK**		
**Predictor**	Unadjusted Odds Ratio	Unadjusted 95% CI	Unadjusted p-value	Unadjusted Odds Ratio	Unadjusted 95% CI	Unadjusted p-value
**PHARMACOKINETIC FACTORS**						
Rifampicin $AUC_{0–24h}$ (for every 10 µg·h/mL)	1·03	0·98 - 1·07	0·26	1·01	0·96 - 1·06	0·70
Rifampicin C_{max} (for every 1 µg/mL)	1·03	0·99 - 1·06	0·12	1·02	0·98 - 1·06	0·27
**Ethambutol $AUC_{0–24h}$ (for every 5 µg·h/mL)**	**1·40**	**1·01 - 1·94**	**0·045**	**1·47**	**1·03 - 2·10**	**0·032**
**Ethambutol C_{max} (for every 1 µg/mL)**	1·40	0·99 - 1·94	0·051	**1·56**	**1·10 - 2·22**	**0·013**
Pyrazinamide $AUC_{0–24h}$ (for every 100 µg·h/mL)	1·16	0·94 - 1·42	0·15	1·23	0·99 - 1·51	0·061
**Pyrazinamide C_{max} (for every 10 µg/mL)**	**1·52**	**1·11 - 2·06**	**0·00792**	**1·69**	**1·21 - 2·35**	**0·00192**
Isoniazid $AUC_{0–24h}$ (for every 5 µg·h/mL)	1·02	0·94 - 1·11	0·62	1·07	0·98 - 1·16	0·15
**Isoniazid C_{max} (for every 1 µg/mL)**	1·17	0·90 - 1·52	0·24	**1·48**	**1·11 - 1·96**	**0·0071**

Supplementary Table 15. Multivariable Logistic Regression Safety Sensitivity Analysis of Pharmacokinetic Factors Including and Excluding Imputed Values. (A) Rifapentine-Moxifloxacin Regimen, (B) Rifapentine Regimen, (C) Control Regimen. Odds ratios, confidence intervals and two-tailed p-values calculated by logistic regression.

**Rifapentine-Moxifloxacin Regimen**	**Main Analysis Including Imputed PK**			**Sensitivity Analysis Excluding Imputed PK**		
**Predictor**	Adjusted Odds Ratio	Adjusted 95% CI	Adjusted p-value	Adjusted Odds Ratio	Adjusted 95% CI	Adjusted p-value
Pyrazinamide AUC_{0-24h} (for every 100 µg·h/mL)	1·23	1·03 - 1·47	0·022	1·24	1·03 - 1·48	0·023
Age (for every 10 years)	1·22	1·06 - 1·41	0·0058	1·19	1·03 - 1·39	0·021
History of liver disease	7·43	1·42 - 54·3	0·022	3·94	0·47 - 33·4	0·17

**Rifapentine Regimen**	**Main Analysis Including Imputed PK**			**Sensitivity Analysis Excluding Imputed PK**		
**Predictor**	Adjusted Odds Ratio	Adjusted 95% CI	Adjusted p-value	Adjusted Odds Ratio	Adjusted 95% CI	Adjusted p-value
Asian Race (relative to Black)	2·44	1·45 - 3·99	0.00051	2·44	1·45 - 3·99	0.00051
History of liver disease	5·74	1·19 - 30·5	0·027	5·74	1·19 - 30·5	0·027

**Control Regimen**	**Main Analysis Including Imputed PK**			**Sensitivity Analysis Excluding Imputed PK**		
**Predictor**	Adjusted Odds Ratio	Adjusted 95% CI	Adjusted p-value	Adjusted Odds Ratio	Adjusted 95% CI	Adjusted p-value
Female sex (relative to male)	1·74	1·17 - 2·56	0·00519	1·74	1·17 - 2·56	0·00519
Xpert MTB/RIF CT (for every 3 CT increase)	1·22	1·05 - 1·42	0·00875	1·22	1·05 - 1·42	0·00875

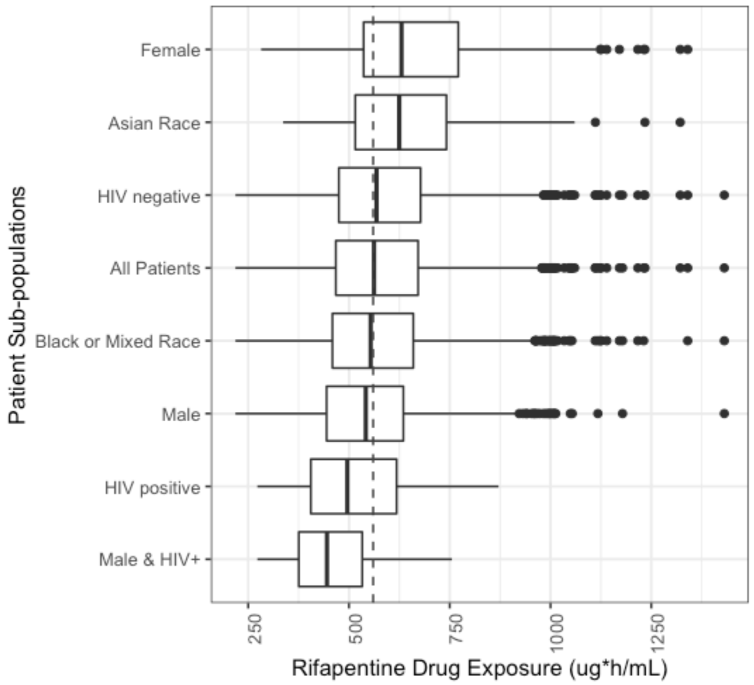

Supplementary Figure 8. All Participants Have the Potential for Low Rifapentine Exposure, but Male Participants and Participants Living with HIV are at Higher Risk of Low Drug Exposure. The dotted line represents the median exposure for all patients, 561 μg•h/mL. Low rifapentine exposure also greatly increases the risk for tuberculosis-related unfavorable outcomes, therefore identifying subpopulations at risk of low rifapentine exposure is important. Although any participant has the potential for low rifapentine exposure, male participants or participants living with HIV have the highest risk of low rifapentine exposure.

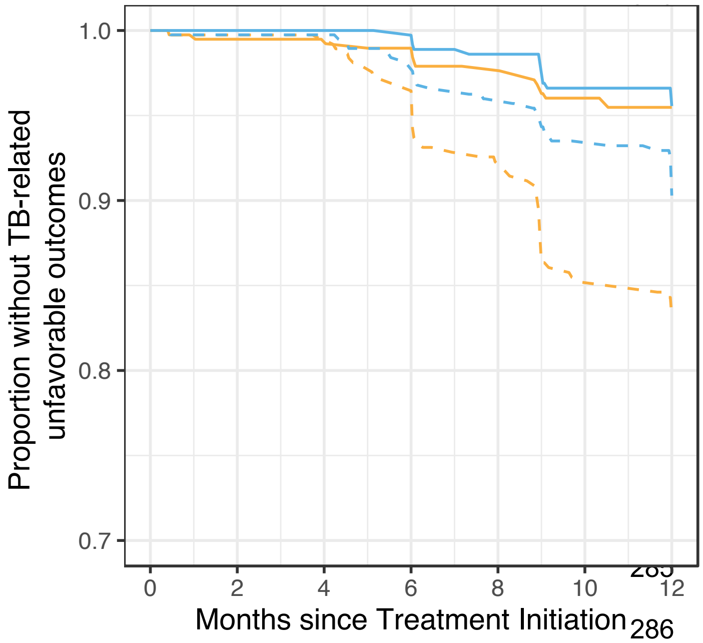

RPT AUC	Regimen	Number at Risk						
Above Median	HPZE	395	380	378	375	366	355	190
Below Median	HPZE	386	375	365	352	328	295	146
Above Median	HPZM	386	373	365	361	349	335	184
Below Median	HPZM	402	384	375	366	351	335	164

Supplementary Figure 9. Rifapentine Exposure is Crucial in Driving Treatment Response. Participants with above median rifapentine exposure (solid lines) have comparable cure rates regardless of receiving the rifapentine regimen (yellow solid) or the rifapentine-moxifloxacin (blue solid) regimen. Participants with below median rifapentine exposure (dotted lines) had markedly improved cure rates with the substitution of moxifloxacin for ethambutol.
